# Retinal pigment epithelium-specific ablation of GPx4 in adult mice recapitulates key features of geographic atrophy in age-related macular degeneration

**DOI:** 10.1038/s41419-024-07150-2

**Published:** 2024-10-19

**Authors:** Kunihiro Azuma, Takafumi Suzuki, Kenta Kobayashi, Masako Nagahara, Hirotaka Imai, Akiko Suga, Takeshi Iwata, Tomoyasu Shiraya, Makoto Aihara, Takashi Ueta

**Affiliations:** 1 Department of Ophthalmology, The Graduate School of Medicine and Faculty of Medicine, The University of Tokyo, Bunkyo Ward, Japan; 2 Department of Ophthalmology, National Center for Global Health and Medicine, Shinjuku Ward, Japan; 3 Section of Viral Vector Development, Center for Genetic Analysis of Behavior, National Institute for Physiological Sciences, National Institutes of Natural Sciences, Okazaki, Aichi Japan; 4 Department of Hygienic Chemistry and Medical Research Laboratories, School of Pharmaceutical Sciences, Kitasato University, Tokyo, Japan; 5 Molecular and Cellular Biology Division, National Institute of Sensory Organs, NHO Tokyo Medical Center, Tokyo, Japan

**Keywords:** Experimental models of disease, Molecular biology

## Abstract

Age-related macular degeneration (AMD) is a leading cause of irreversible vision loss in the elderly population, particularly the late-stage of dry AMD known as geographic atrophy (GA), lacks effective treatment options. Genetic mouse models of AMD have revealed the significance of impaired lipid metabolism and anti-oxidative capacity in early/intermediate stage of AMD, but remains unclear in GA that severely damages visual function. Here, to investigate the potential relevance of peroxidized lipids in RPE for late-stage dry AMD, GPx4^fl/fl^ mice underwent subretinal injections of RPE-specific AAV-Cre vector or control AAV vector. RPE-specific GPx4 deficiency led to rapid RPE degeneration resembling key features of late-stage dry AMD, including preceding loss of RPE cell polarity, accumulation of acrolein, malondialdehyde, and 4-hydroxynonenal, photoreceptor loss, lipofuscin-laden subretinal melanophage infiltration, and complement activation. Treatment with α-tocopherol and ferrostatin-1 mitigated RPE degeneration, and shrunk mitochondria were observed in GPx4 deficient mice, suggesting involvement of ferroptosis. Unexpectedly, necrostatin-1s, an inhibitor of necroptosis, also ameliorated RPE degeneration, and activation of RIP3 and MLKL along with inactivation of caspase-8 was observed, indicating crosstalk between ferroptosis and necroptosis pathways. Our findings shed light on the intricate mechanisms underlying RPE degeneration in AMD and highlight GPx4/lipid peroxidation as potential therapeutic targets. RPE-specific ablation of GPx4 in mice provides a valuable tool for further elucidating the interplay between lipid peroxidation, cell death pathways, and AMD pathogenesis, offering new insights for preclinical research and therapeutic development targeting GA.

## Introduction

Retinal pigment epithelium (RPE) is a monolayer of pigmented epithelial cells positioned between photoreceptor outer segments (OSs) and the choriocapillaris of the choroid. RPE cells are highly polarized, and have apical microvilli enveloping the OSs of photoreceptors and basal infoldings in the baso-lateral membrane [1, 2]. RPE cells play multifaceted roles in vision, including participating in visual cycle, forming the blood-retinal barrier, supplying nutrients and growth factors to photoreceptors and choroid, and protecting the retina from oxidative stress [2–5]. A key task of RPE cells involves phagocytosing and metabolizing 10% of photoreceptor OS daily [3, 6], wherein 90% of OSs comprise phospholipids, and the rest is cholesterol and glycolipids. Importantly, OSs are enriched by four folds with polyunsaturated fatty acid (PUFA) that are highly susceptible to peroxidation upon light exposure compared to other plasma membrane [2, 7, 8], necessitating mechanisms to detoxify peroxidized phospholipids for the homeostasis of RPE and vision.

Pathological alterations in the RPE invariably impair visual function and can lead to permanent blindness, given the absence of regenerative capacity in mammalian RPE cells [9]. Age-related macular degeneration (AMD) is a major cause of blindness worldwide. The onset of AMD manifests in early and intermediate stages characterized by the formation of extracellular lipid depositions called drusen and subretinal drusenoid deposits around the RPE, while maintaining relatively preserved vision. Advanced AMD stages, associated with significant visual loss, encompass neovascular (wet) AMD (~15%) and atrophic (dry) AMD, also known as geographic atrophy (GA) (85%) [10, 11]. Notably, while effective therapies exist for wet AMD, no established treatment exists for dry AMD, although complement inhibitors have been debated [12]. The etiology of AMD, though not fully elucidated, implicates oxidative stress, impaired lipid metabolism, and inflammation [1, 13, 14]. Genetically engineered mouse models mirroring abnormal lipid metabolism and impaired anti-oxidative defense have provided insights into AMD development, particularly in early and intermediate stages [13, 15]. However, their relevance to late-stage dry AMD or GA, particularly concerning endogenously produced peroxidized lipids in the RPE, remains obscure.

Glutathione peroxidase 4 (GPx4), an antioxidant enzyme ubiquitously expressed, has a distinctive role in directly detoxifying peroxidized phospholipids within cell membranes and thwarting ferroptosis, a lipid peroxidation-induced form of non-apoptotic cell death [16]. Conventional knockout (KO) of GPx4 results in early embryonic lethality [17], while conditional KO (cKO) mice with GPx4 deficiency in various cell types exhibits loss of homeostasis and cell death [18–21], except in select cell types including myeloid cells [22] and vascular endothelial cells [23].

In this context, we developed RPE-specific cKO mice for GPx4 and scrutinized their degenerative phenotypes to unravel the significance of regulating peroxidized lipids for RPE in vivo. Our study furnishes direct evidence implicating endogenous lipid peroxidation products in RPE in inducing pathological features reminiscent of GA, with involvement of ferroptosis and necroptosis.

## Materials and methods

### Construction of AAV vectors

**Fig. 1:**
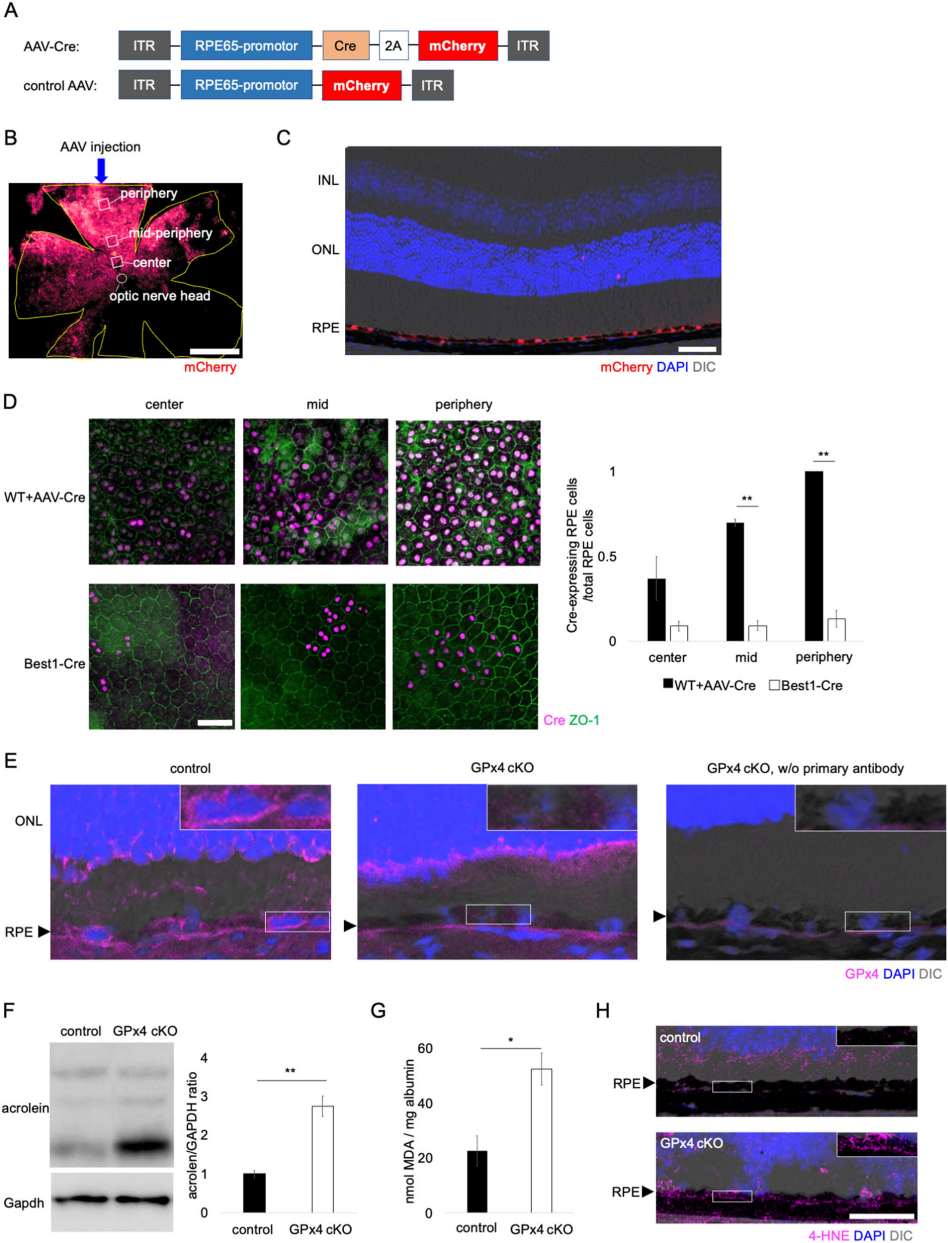
Construction of AAV vectors. **A** Structures of AAV-Cre vector and control AAV vector used in this study. **B** mCherry expression in the WT RPE observed on flatmount after a subretinal injection of control AAV. Central (close to the optic nerve head), mid-peripheral (750 µm from the optic nerve head), and peripheral (1500 µm from the optic nerve head) regions (0.12 mm^2^ each) were examined to evaluate topographical variations. Scale bar, 1 mm. **C** mCherry expression in the RPE was confirmed after a subretinal injection of AAV-Cre in WT mice. Scale bar, 50 µm. **D** Ratio of Cre-expressing RPE cells to total RPE cells was compared between AAV-Cre-injected WT mice and Best1-Cre mice. *n* = 3–4 per group. Scale bar, 50 µm. **E** GPx4 ablation in the RPE was confirmed in GPx4 cKO mice. The sections were partially de-melanized. The insets are a higher magnification of the selected areas of the RPE layer. Black arrowheads indicate RPE layer. Scale bar, 20 µm. **F** Accumulation of acrolein, an aldehyde produced by lipid peroxidation, in the RPE-choroid was compared on western blot between GPx4 cKO and control mice. *n* = 4 per group. **G** Accumulation of MDA in the RPE-choroid was compared between GPx4 cKO and control mice. *n* = 4–5 per group. **H** 4-HNE accumulation in the RPE was compared between GPx4 cKO and control mice after 12 days of subretinal AAV injections. The insets are a higher magnification of the selected areas of the RPE layer. Black arrowheads indicate RPE layer. Scale bar, 50 µm. Data are presented as a mean ± SEM.

pAAV-CAG-EGFP (addgene, plasmid #37825) was modified by cutting at BamHI/HindIII to replace EGFP with mCherry sequence (addgene, plasmid #58909) and cutting at NdeI/XbaI to replace CAG promoter with RPE65 promoter sequence (Vigene Biosciences), resulting in the creation of pAAV-RPE65-mCherry. Separately, pAAV-CAG-EGFP (addgene, plasmid #37825) was modified by cutting at DraIII/EcoRV to replace ITR and CAG-EGFP with the sequence of ITR and RPE65-Cre-2A-mCherry. Cre-2A-mCherry sequence was from pAAV-ProC3-Cre/mCherry (addgene, plasmid #126006). AAV_DJ_ vectors were made with AAV Helper Free Packaging System (Cell Biolabs, San Diego, CA, USA) as described previously (Fig. 1A) [24]. RT-qPCRs were run to determine virus titers. Those AAV vector solutions were diluted with PBS to 1.0 × 10^13^ vg/ml for subretinal injection.

### Animal experiments

All animal experiments follow the Association for Research in Vision and Ophthalmology Statement for the Use of Animals in Ophthalmic and Vision Research. Protocols for animal procedures underwent reviewed and approval by the Institutional Animal Research Committee of the University of Tokyo. Mice were housed in cages with standard conditions, including access to food and water, and maintained under 12-hour light/dark cycle at 22 °C.

GPx4^fl/fl^ mice, obtained from Jackson Laboratory (strain #:027964) [25]. are C57BL/6J background and free of *rd8* mutation. The mice harbor loxP sites flanking exons 2–4 of the GPx4 gene. To establish RPE-specific GPx4 conditional KO (GPx4 cKO) mice, 8-week-old GPx4^fl/fl^ mice were randomly chosen, and underwent subretinal injection of AAV_DJ_-RPE65-Cre-2A-mCherry (AAV-Cre) at 2.0 × 10^10^ vg/eye using a micro-syringe (MS-NE05, Ito corporation, Shizuoka, Japan) and a 33 G needle (Ito corporation) under a surgical microscope (Universal S3, Zeiss, Carl Zeiss, Jena, Germany). Just before the subretinal injection, anterior chamber paracentesis using the tip of a 27 G/30 G needle was performed to make a space in the eye for the following injection of AAV solution. After subretinal injection, a coverslip was placed on the cornea and the fundus was observed. Success of the subretinal injection was judged based on an appearance of retinal detachment. Success rate was favorable (80–90%), and the other unsuccessful eyes were excluded. For control, the other GPx4^fl/fl^ mice received subretinal injection of AAV_DJ_-RPE65-mCherry (control AAV) at 2.0×10^10^ vg/eye. Mice were sacrificed 12–14 days or 42 days post-AAV injections for analyses.

Best1-Cre mice were obtained from Jackson Laboratory (strain #017557).

To investigate the effect of vitamin E (Vit E), mice were maintained on standard rodent diet CE-2 supplemented with DL-α-tocopherol acetate (1000 IU/kg) (CLEA Japan) for 2 weeks, one week before and after AAV injections.

Ferrostatin-1 (Fer-1), necrostatin-1s (Nec-1s), and Z-VAD-FMK (ZVAD, a pancaspase inhibitor) to GPx4 cKO mice was administered via both intraperitoneal and intravitreal routes to maximize the pharmacological effects. Intraperitoneal injections were administered daily from day 10–13 post-AAV injections at dosages of 4.0 mg/kg, 1.6 mg/kg, and 4 mg/kg for Fer-1, Nec-1s, and ZVAD, respectively, with a vehicle control was 2%DMSO in PBS. Intravitreal injections were conducted once on day 13 post-AAV injections with 2.0 µl of 100 µM Fer-1, 300 µM Nec-1s, and 300 µM ZVAD in PBS with 1%DMSO vehicle.

Spectral domain-optical coherence tomography (SD-OCT) was performed using OCT Bi-µ (KOWA, Tokyo, Japan). Fundus photography was conducted using Micron IV (Phoenix-Micron Inc., Oregon, USA).

### Immunohistochemistry, evaluation of RPE damage, and autofluorescent imaging

For histological analysis, enucleated eyeballs were fixed in 4% paraformaldehyde for 2 h and dehydrated in 35% sucrose in 4 °C overnight. Enucleated eyes were cryosectioned at −20 °C (10μm thickness). For melanin bleaching, sections were soaked in 10% hydrogen peroxide in PBS at 60 °C for 30 min. After blocking using Blocking One Histo (NACALAI TESQUE, INC, Kyoto, Japan) for 10 min, slides were incubated overnight at 4 °C with primary antibodies, and then incubated with secondary antibodies and 4,6-diamidino-2-phenylindole (DAPI) (Sigma-Aldrich, Burlington, MA) for 1 h at room temperature. The primary and secondary antibodies used are shown in Supplementary Table S1. The sections were coverslipped with mounting medium (Vector Laboratories, Burlingame, CA), and observed under a confocal microscope (Zeiss LSM880, Zeiss).

Immunohistochemistry of RPE flatmounts were carried out as described previously [26]. Briefly, enucleated mice eyes were fixed in 4% paraformaldehyde for 1 h, anterior part of the eye, lens, and retina were removed, then the RPE/choroid was dissected into flatmounts. They were incubated with primary antibodies overnight at 4 °C, then incubated with secondary antibodies for 1 h at room temperature, and coverslipped with mounting medium (Vector Laboratories, Burlingame, CA). The flatmouted RPE samples were subjected to grading based on RPE damage scale proposed recently [27]. A subretinal AAV injection resulted in the gene transfer to the RPE, primarily affecting approximately half of the total RPE area around the injection site (Fig. 1B). Consequently, our evaluations were performed on the same side as the injection. Topographical variations were assessed at the central (close to the optic nerve head), middle (750 µm from the optic nerve head), and peripheral (1500 µm from the optic nerve head) regions (0.12 mm^2^ each) (Fig. 1B). Morphometric assessments of the RPE cells were performed using at least 3 eyes of 3 mice per group, and in each eye, predefined 3 center, 3 middle, and 3 peripheral areas were assessed. The assessments were conducted in blinded fashion, and the evaluator (K.A.) was masked to which group each sample was allocated.

Autofluorescent images of mouse retinal sections were captured by 450/490 for excitation and 500/550 for emission wavelength under a confocal microscope (Zeiss LSM880, Zeiss).

### Transmission electron microscope (TEM)

Tissues were prefixed by 2% PFA + 2% glutaraldehyde in 0.1 M cacodylate buffer pH7.4 overnight at 4 °C. After washing with 0.1 M cacodylate buffer, they were postfixed by 2% osmium tetroxide in 0.1 M cacodylate buffer for 3 h at 4 °C. The tissues were dehydrated through increasing concentrations of ethanol, infiltrated using propylene oxide, and embedded in Quetol-812 (Nisshin EM, Tokyo, Japan).

Ultrathin sections of 70 nm thickness were made using an ultramicrotome (Ultracut UCT, Leica, Vienna, Austria). Electronic staining was performed by 2% uranyl acetate for 15 min at room temperature and lead stain solution (Sigma-Aldrich) for 3 min at room temperature. TEM images were captured using a transmission electron microscope (JEM-1400Plus, JEOL Ltd., Tokyo, Japan) via a charge-coupled device camera (EM-14830RUBY2, JEOL Ltd.).

### Western blotting

Tissues were homogenized in RIPA buffer (Thermo Fisher Scientific, Waltham, MA) with protease inhibitor cocktail (nacalai tesque). The proteins were electrophoresed with NuPage Bis-Tris Gels (Thermo Fisher Scientific) at 200 V, then transferred into PVDF membrane. The membranes were blocked with Blocking One (nacalai tesque) for 10 min, incubated with primary antibodies overnight at 4 °C, washed, incubated with HRP-conjugated secondary antibodies for 1 h, and washed. Blot images were captured using ImageQuant LAS 4000 mini-camera system (GE Healthcare, Chicago, IL). The primary and secondary antibodies were shown in Table S1.

Soluble and insoluble fractions of proteins were extracted as reported previously [28, 29]. Tissues were homogenized in RIPA buffer with protease inhibitor and phosphatase inhibitor cocktail. The lysates were centrifuged at 15,000× *g* for 20 min at 4 °C. The supernatants were collected as the soluble protein fraction. For insoluble protein fraction, the pellet was resuspended in RIPA buffer supplemented with 8 M urea (Fujifilm Wako, Tokyo, Japan) and protease and phosphatase inhibitor (Sigma-Aldrich), and then sonicated briefly.

### Thiobarbituric Acid Reactive Substances (TBARS) measurement

TBARS (TCA method) assay kit (Cayman Chemical, Ann Arbor, MI) was used for measuring Malonaldehyde-bis-DimethylAcetal (MDA) following the manufacture’s protocol. The fluorescence was read using a plate reader (2030 ARVO X3, PerkinElmer, Waltham, MA) at an excitation wavelength of 530 nm and an emission wavelength of 550 nm. Fluorescence readings were converted to concentration values based on the standard curve, and then adjusted to the albumin concentration measured using BCA assay kit (Thermo Fisher Scientific, Waltham, MA).

### Statistics

Statistical analyses were performed by EZR [30] (Saitama Medical Center, Jichi Medical University), which is based on R 4.0.3 (The R Foundation for Statistical Computing). EZR is a modified version of R commander designed to add statistical functions frequently used in biostatistics. In experiments, biological triplicate and technical duplicate were ascertained, and data were analyzed in a blinded fashion. Normal distribution and homogeneity of variance were confirmed by Shapiro–Wilk and Levene’s test, respectively. Sample size was determined by statistical power analysis with α = 0.05 and β = 0.2. No data or animals were not excluded as long as the subretinal injections were successfully performed. Data are presented as a mean ± SEM. Unpaired Student’s *t* test or one-way ANOVA followed by Tukey’s post hoc test was used for numerical data analysis. A *P* value of <0.05 Values was considered statistically significant. Asterisks mark *P* values ≤ 0.05 (*) or ≤0.01 (**).

## Results

### Generation of GPx4 cKO mice

RPE-specific existence of AAV-Cre vector-derived mCherry expression was confirmed in wild-type (WT) mice following subretinal injection of AAV-Cre vector (Fig. 1C). In this study we used AAV-Cre vector in GPx4^fl/fl^ mice to investigate the effects of GPx4 deletion in the adult mice. While Best1-Cre mice are commonly used for creating RPE-specific cKO mice, Cre expression initiates as early as at least postnatal day 10 (P10) [31]. Additionally, Cre expression in Best1-Cre mice exhibits considerable variability, with 10–90% of total RPE cells expressing Cre due to individually different epigenetic silencing mechanisms [32, 33]. In our experiments, we found that Cre expression using AAV-Cre vector was more consistent and at higher levels compared to using Best1-Cre mice (Fig. 1D). In GPx4 cKO mice, depletion of GPx4 protein specifically in the RPE was confirmed after AAV-Cre injections (Fig. 1E). As a result, a significant increase in acrolein and MDA, lipid peroxidation aldehydes related to AMD [34–38], in the RPE-choroid tissues was detectable (Fig. 1F, G), despite Cre gene transfer occuring in only approximately half of the total RPE area. Furthermore, immunohistochemistry confirmed that another aldehyde 4-Hydroxynonenal (4-HNE) accumulates in the RPE of the GPx4 cKO mice as early as 12 days after AAV-Cre injections (Fig. 1H, Supplementary Fig. S1).

### GPx4 deficiency leads to RPE degenerations

**Fig. 2:**
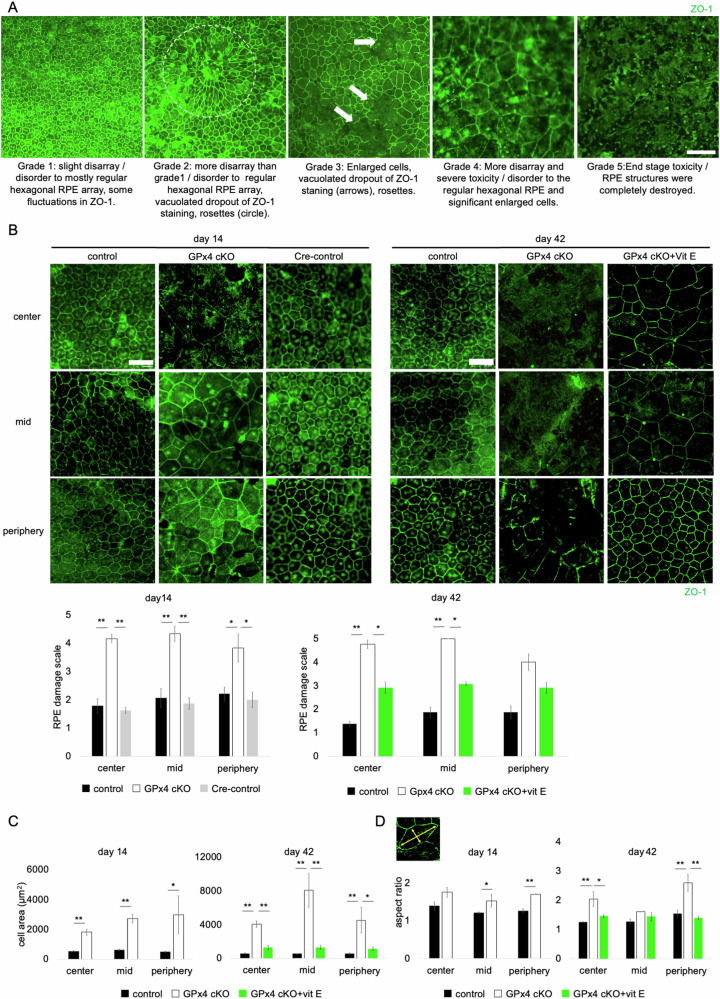
RPE degeneration in GPx4 cKO mice. **A** RPE degeneration grading based on a previously proposed scale. Scale bar, 100 µm. **B** 14 days after AAV injections, RPE degeneration grade was compared among control (GPx4^fl/fl^ mice injected with control AAV), GPx4 cKO (GPx4^fl/fl^ mice injected with AAV-Cre), and Cre-control mice (WT mice injected with AAV-Cre). 42 days after AAV injections, RPE degeneration grade was compared among control, GPx4 cKO, and vit E-supplemented GPx4 cKO mice. *n* = 4–6 per group. Scale bar, 50 µm. **C** 14 days after AAV injections, RPE cell area was compared between control and GPx4 cKO mice. *n* = 3–4 per group. 42 days after AAV injections, RPE cell area was compared among control, GPx4 cKO, and vit E-supplemented GPx4 cKO mice. *n* = 3–5 per group. **D** 14 days after AAV injections, aspect ratio of RPE cells was compared between control and GPx4 cKO mice. 42 days after AAV injections, aspect ratio of RPE cells was compared among control, GPx4 cKO, and vit E-supplemented GPx4 cKO mice. *n* = 3–5 per group. Data are presented as a mean ± SEM.

To assess RPE degeneration in GPx4 cKO mice, the RPE damage scale proposed in a recent study [27] was used (Fig. 2A). The RPE of GPx4 cKO mice exhibited rapid degeneration evident as early as 14 days after the AAV-Cre injections, in all areas of different distances from the optic nerve head (i.e., center, middle, or periphery) (Fig. 2B). The degree of degeneration was mildest in the RPE of control mice (GPx4^fl/fl^ mice receiving control AAV) and Cre-control mice (WT mice receiving AAV-Cre) (Fig. 2B). As expected, supplementation of α-tocopherol (vit E), a lipophilic antioxidant, significantly ameliorated RPE degeneration in GPx4 cKO mice, confirming the lipid peroxidation-dependent mechanisms underlying the degeneration in GPx4-deficient RPE cells (Fig. 2B). Additionally, since healthy RPE cells exhibit a uniform regular hexagonal morphology, we compared the area and aspect ratio of RPE cells between control and GPx4 cKO mice. The results revealed that the RPE cells of GPx4 cKO mice were enlarged (Fig. 2C) and irregularly shaped (Fig. 2D), with worsening progressively at day 14 and 42 after Cre gene transfer, the phenomenon was significantly suppressed by vit E supplementation (Fig. 2C, D).

### GPx4 cKO mice mimic clinicopathological features of human AMD

**Fig. 3:**
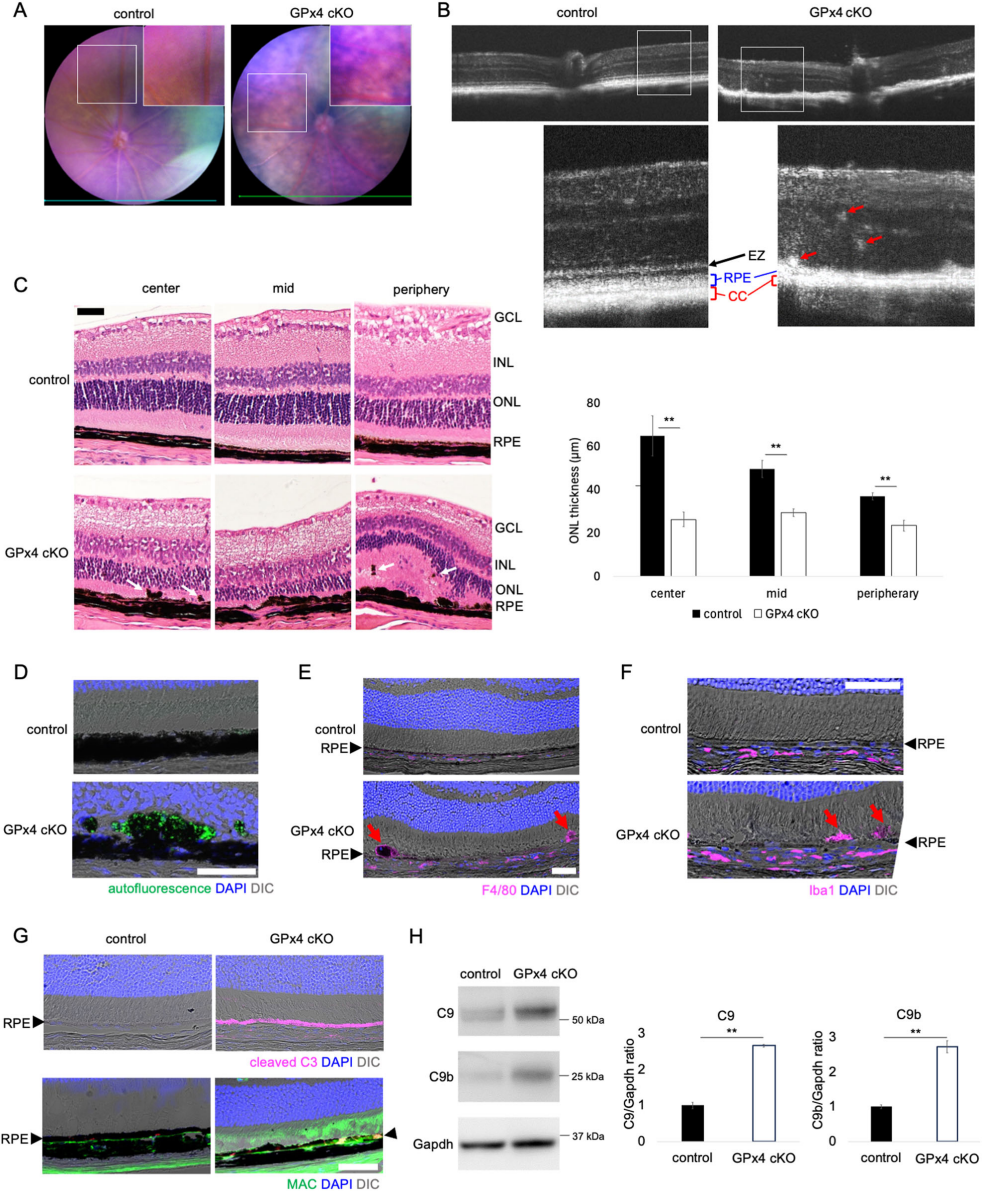
GPx4 cKO mice mimicking clinicopathological features of AMD. **A** Retinal fundus images of control (GPx4^fl/fl^ mice injected with control AAV) and GPx4 cKO (GPx4^fl/fl^ mice injected with AAV-Cre) mice 28 days after injections of AAV vectors. The insets are a higher magnification of the selected area to clearly show mottled hypopigmentation of the GPx4 cKO mice compared to uniform texture of the control mice. **B** SD-OCT images of control and GPx4 cKO mice 14 days after injections of AAV vectors. The insets show a higher magnification of the area in the corresponding white rectangle, showing hyperreflective foci (red arrows). EZ, ellipsoid zone. CC, choriocapillaris. **C** ONL thickness was compared on Hematoxylin-eosin staining between control and GPx4 cKO mice 14 days after injections of AAV vectors. White arrows indicate pigment containing cells in the neural retina. *n* = 6 per group. Scale bar, 50 µm. **D** Autofluorescence of subretinal and intraretinal pigment containing cells in GPx4 cKO mice 14 days after AAV injections. Scale bar, 50 µm. **E** F4/80 expression of subretinal pigment containing cells in GPx4 cKO mice (red arrows). Black arrowheads indicate RPE layer. Scale bar, 50 µm. **F** Iba1 expression of subretinal pigment containing cells in GPx4 cKO mice (red arrows) 13 days after AAV-Cre injections. Black arrowheads indicate RPE layer. Scale bar, 50 µm. **G** Activation of complement component C3 in the RPE and MAC deposition in and around the RPE of GPx4 cKO mice 13 days after injections of AAV-Cre vectors. Black arrowheads indicate RPE layer. Scale bar, 50 µm. **H** Abundance of zymogen C9 and C9b fragment in the RPE-choroid was compared on western blot between GPx4 cKO and control mice 14 days after injections of AAV vectors. *n* = 5 per group. Data are presented as a mean ± SEM.

Retinal fundus images of the GPx4 cKO mice revealed mottled hypopigmented lesions compared to control mice after 28 days of AAV-Cre injections, while no apparent abnormality was observed in the fundus of control mice (GPx4^fl/fl^ mice receiving control AAV) (Fig. 3A). SD-OCT of GPx4 cKO mice showed loss of the ellipsoid zone of the photoreceptors, irregular and thinner RPE line, a thinner choriocapillaris layer, and hyperreflective foci 14 days after AAV-Cre injections (Fig. 3B). Hematoxylin-eosin staining of the retinal sections 14 days after AAV-Cre injections displayed reduced outer nuclear layer (ONL) thickness (i.e., loss of photoreceptors), loss of RPE cells, and localization of pigment-containing cells in the subretinal space and neural retina (Fig. 3C). The subretinal and intraretinal pigmented cells in GPx4 cKO mice exhibited higher autofluorescence, indicative of lipofuscin accumulation (Fig. 3D). In AMD, hyperreflective foci on SD-OCT serve a biomarker for progression to the late stage of AMD, consisting of melanosome/melanolipofscin-laden mononuclear phagocytes, termed melanophages [39]. To further characterize pigment-containing cells in GPx4 cKO mice, ocular sections were examined via immunohistochemistry after partial de-melanization due to the strong black pigment of C57BL6/J strain blocking fluorescent signals. The results confirmed that subretinal pigmented cells were positive for F4/80 and Iba1, suggesting melanophage localization in the subretinal space of GPx4 cKO mice (Fig. 3E, F). Accumulation of lipofuscin-laden pigmented microglia/macrophages in the subretinal space have also been observed in other AMD mouse models [40–42] and aged WT mice [43]. Furthermore, complement activation in the RPE is another key feature of AMD in humans [44, 45] and also found in some mouse models [42, 46]. In GPx4 cKO mice, activation of complement component C3 (cleaved C3) was specifically observed in the RPE (Fig. 3G), and membrane attack complex (MAC) deposition was observed in and around the RPE (Fig. 3G). In addition, zymogen C9 (70 kD) is proteolytically cleaved at a specific site to induce C9 polymerization [47]. The 25 kD product C9b, which is the carboxyl-terminal fragment of C9 capable of disturbing membrane potential, was identified as a marker of membrane attack complex (MAC) formation in the NaIO_3_-induced dry AMD (GA) model [46]. The RPE-choroid of GPx4 cKO mice contained significantly increased levels of both C9 and C9b (Fig. 3H).

### Ultrastructure of RPE degeneration in GPx4 cKO mice

**Fig. 4:**
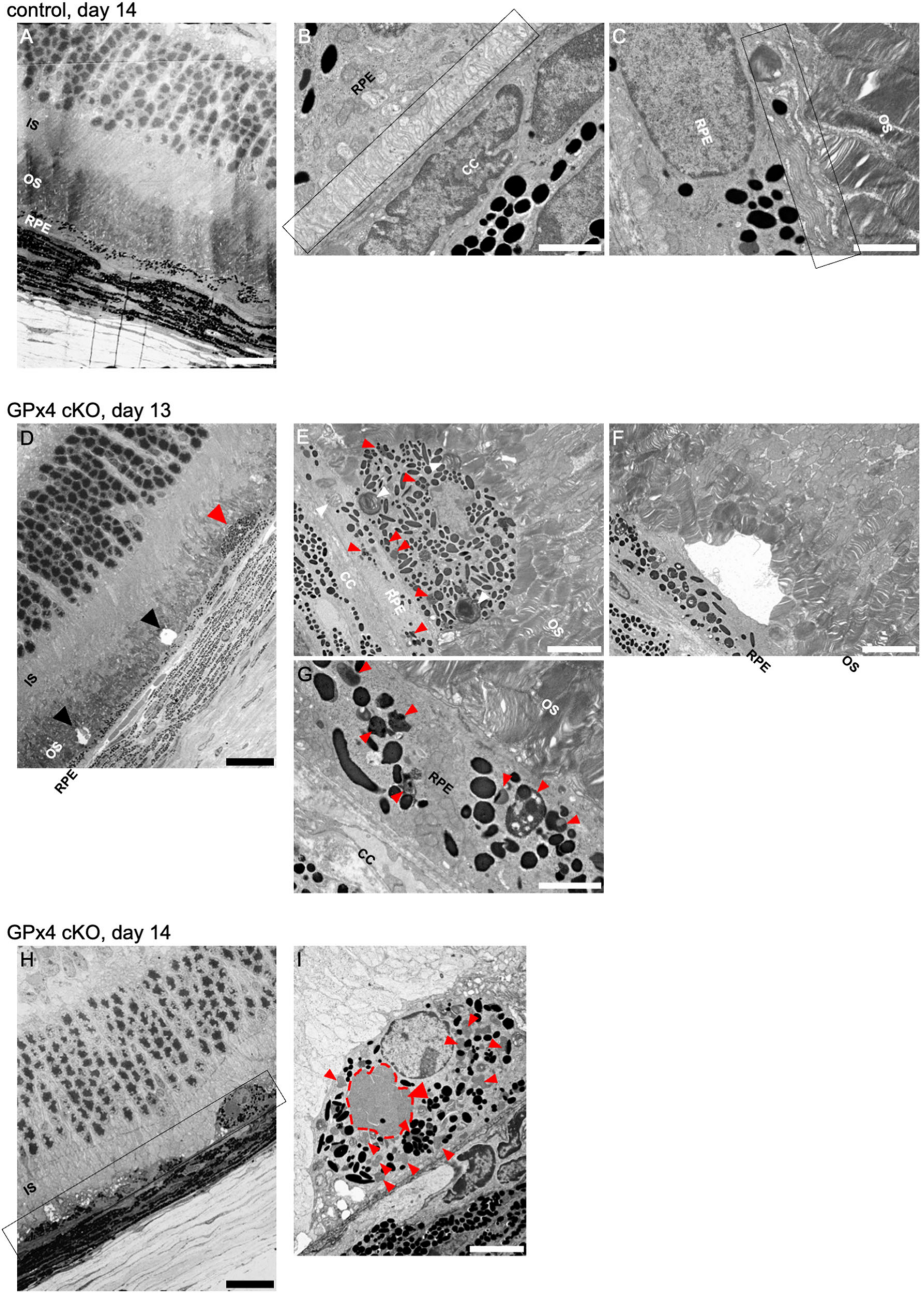
Ultrastructure of RPE degeneration in GPx4 cKO mice. **A** A lower magnification TEM image of a control mouse retina 14 days after injections of control AAV vector to GPx4^fl/fl^ mice. Scale bar, 20 µm. **B** At higher magnification, normal basal infoldings of RPE (area inside rectangle), and **C** apical microvilli enveloping photoreceptor OS (area inside rectangle) are observed. Scale bar, 2 µm. **D** A lower magnification TEM image of a GPx4 cKO mouse retina 13 days after AAV-Cre injections to GPx4^fl/fl^ mice. A subretinal pigment-containing cell (red arrowhead). Subretinal voids (black arrowheads). Scale bar, 20 µm. **E** At higher magnification, melanolipofuscin (red arrowheads), and undigested OS particles (white arrowheads) are observed in pigment-containing subretinal cells and RPE cells. Scale bar, 5 µm. **F** Subretinal voids observed in GPx4 cKO mice. Scale bar, 5 µm. **G** Loss of apical microvilli and basal infoldings of RPE cells. Melanolipofuscin (red arrowheads). Scale bar, 2 µm. **H** A lower magnification TEM image of a GPx4 cKO mouse retina 14 days after AAV-Cre injections to GPx4^fl/fl^ mice. The area surrounded by a rectangle indicates loss of OS and RPE cells. Scale bar, 20 µm. **I** At higher magnification, pigment containing subretinal cells contain significant volume of lipofuscin and melanolipofuscin, some of which are indicated by red arrowhead and a dashed line. Scale bar, 5 µm. IS inner segment, OS outer segment, RPE retinal pigment epithelium, CC choriocapillaris.

TEM was employed to further elucidate the ultrastructure of RPE degeneration in GPx4 cKO mice. In control eyes, normal thickness of photoreceptor OSs (Fig. 4A), basal infoldings (Fig. 4B) and apical microvilli at the interface with OSs (Fig. 4C) were observed, indicating well-polarized structures of RPE cells. Conversely, in GPx4 cKO mice, a thinner OS layer (Fig. 4D), subretinal pigment-containing cells (Fig. 4D, E), localized voids in the subretinal space (Fig. 4F), and loss of polarized structures of RPE cells including microvilli and basal infoldings (Fig. 4G) were observed 13 days after AAV-Cre injections. At this stage, abundant formation of melanolipofuscin were observed in the RPE and in the subretinal melanin-containing cells (Fig. 4E, G). RPE degeneration progressed rapidly, and at 14 days after AAV-Cre injections, OSs and RPE cells were lost (Fig. 4H). Consistent with the findings of subretinal melanophages on immunohistochemistry (Fig. 3D–F), subretinal pigment-containing cells were filled with lipofuscin and melanolipofuscin (Fig. 4I).

### Inhibitors of ferroptosis and necroptosis ameliorated RPE degeneration caused by GPx4 deficiency

**Fig. 5:**
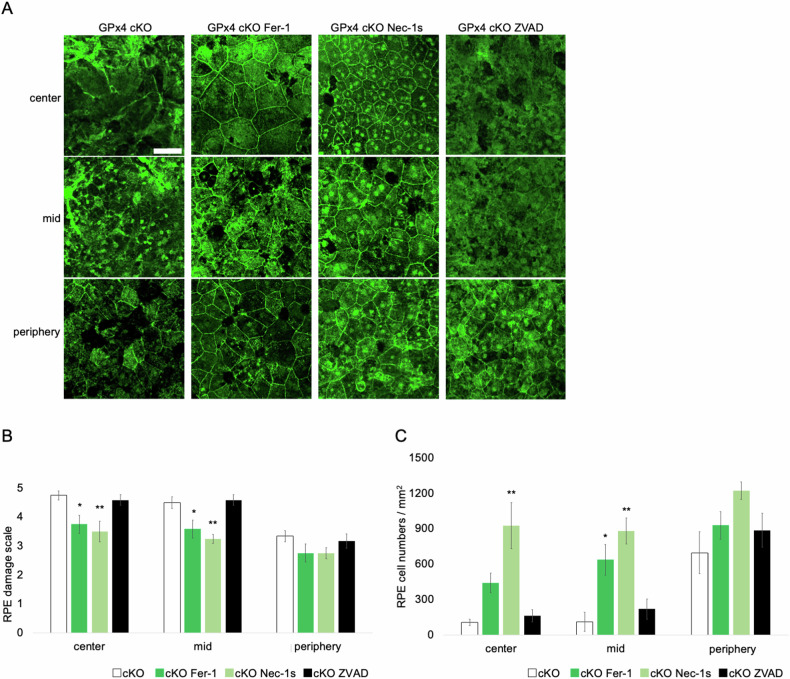
Effects of cell death inhibitors on RPE degeneration in GPx4 cKO mice. **A** RPE degeneration was compared among vehicle-, Fer-1-, Nec-1s-, and ZVAD-treated GPx4 cKO mice, based on **B** the grading scale and **C** the number of remaining RPE cells 14 days after AAV-Cre injections. *n* = 6 per group. Scale bar, 50 µm. Data are presented as a mean ± SEM.

To assess the modes of RPE cell death observed in GPx4 cKO mice, GPx4 cKO mice were treated with inhibitors for ferroptosis (Fer-1), necroptosis (Nec-1s), or apoptosis (ZVAD). Unexpectedly, we observed that not only Fer-1 but also Nec-1s significantly ameliorated the RPE degeneration in terms of the grade assessment and the number of remaining RPE cells, while ZVAD did not have significant effects (Fig. 5A–C). In control mice, the same treatments did not significantly affect the morphology of the RPE cells (Supplementary Fig. S2).

**Fig. 6:**
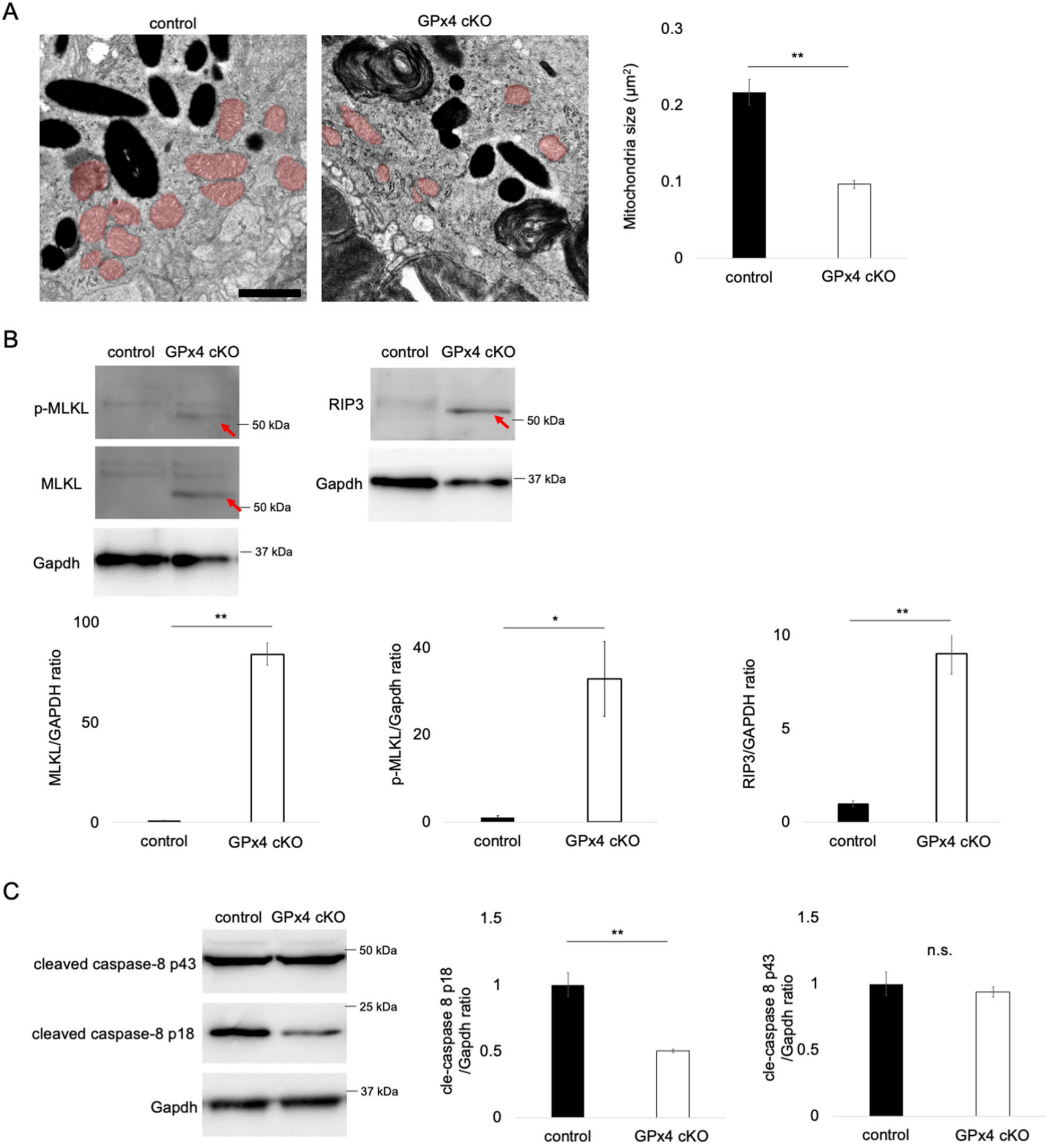
Implications of ferroptosis and necroptosis for RPE degeneration in GPx4 cKO mice. **A** Comparison of mitochondria area (shaded in light red) in the RPE between GPx4 cKO and control mice. *n* = 6 per group. Scale bar, 1 µm. **B** Abundance of MLKL, p-MLKL, and RIP3 in detergent-insoluble fraction samples of RPE-choroid was compared on western blot between GPx4 cKO and control mice 12 days after injections of AAV-Cre and control AAV vectors, respectively. *n* = 4 per group. **C** Abundance of cleaved caspase-8 in the RPE-choroid was compared on western blot between GPx4 cKO and control mice 12 days after injections of AAV-Cre and control AAV vectors, respectively. *n* = 5 per group. Data are presented as a mean ± SEM.

As GPx4 is the master inhibitory regulator of ferroptosis, the amelioration by Fer-1 (Fig. 5) and also by vit E (Fig. 2B–D) were in line with our expectation. Moreover, we observed shrunk mitochondria on TEM, confirming another characteristic ferroptosis phenotype [48, 49] in GPx4 cKO RPE cells (Fig. 6A). Nec-1s is an improved reagent from Nec-1 as Nec-1 is the inhibitor for both RIP1 and indoleamine 2,3-dioxygenase (IDO). Besides, it has been reported that Nec-1 can inhibit both ferroptosis and necroptosis, while Nec-1s is a specific inhibitor of necroptosis [20, 50]. The results suggest involvement of both ferroptosis and necroptosis in the RPE cell loss of GPx4 cKO mice.

To clarify necroptosis in RPE degeneration caused by GPx4 deficiency, the levels of key molecules for the necroptosis activation were investigated in the RPE-choroid samples. Assembly of necrosome comprising phosphorylated RIP1 (p-RIP1)/p-RIP3/p-MLKL is the critical step for the execution of necroptosis. Because of the amyloid structure of the necrosome, translocation of RIP1/RIP3/MLKL into detergent-insoluble fractions is important for necroptosis signal transduction [28, 29, 51, 52]. In the RPE-choroid samples from GPx4 cKO mice, a significant upregulation of RIP3, MLKL, and p-MLKL was observed in the insoluble fraction (Fig. 6B). On the other hand, RIP3 in the soluble fraction was decreased (Supplementary Fig. S3A) and no apparent change in the levels of MLKL and p-MLKL was observed (Supplementary Fig. S3B). These results indicated necrosome formation and activation of necroptosis pathway.

Furthermore, caspase-8 inactivation is a prerequisite for necroptosis activation [53, 54]. In the RPE-choroid of GPx4 cKO mice, the active fragment p18 of caspase-8 was significantly decreased while there was not a significant change in the levels of intermediate fragment p43 (Fig. 6C), which further substantiated the pro-necroptotic conditions in the RPE-choroid tissue of GPx4 cKO mice.

## Discussion

The current study elucidated the crucial role of GPx4 in the RPE in mice, revealing that RPE-originated lipid peroxidation leads to RPE degeneration resembling key features of the late stage of dry AMD. Animal models are indispensable in medical research, aiding in the understanding and treatment of human diseases. In the case of wet AMD, the development of laser-induced choroidal neovascularization model was pivotal for the establishment of anti-VEGF therapy. Similarly, various gene-modified mice related to lipid metabolism have been reported to recapitulate age-related early and intermediate changes of AMD [13, 15, 55, 56]. Additionally, deficiency of anti-oxidative enzymes, including SOD1 [57], SOD2 [58], and Nrf2 [59, 60] has been linked to early and intermediate AMD phenotypes.

However, the roles of these models in the development of GA, the late-stage manifestation of dry AMD, have remained unclear. Previous models take 6–12 months, often up to 2 years, to sufficiently develop early/intermediate AMD phenotypes, such as drusen and subretinal drusenoid deposits. While these models underscore the importance of age in AMD pathogenesis, their utility as preclinical animal models may be limited. In contrast, the laser-induced choroidal neovascularization model for wet AMD can be applicable regardless of the mouse age, and it takes only one week from laser irradiation to the assessment of neovascularization. In the late stage of dry AMD, the scarcity of clinically relevant animal models has posed a significant challenge, leading researchers to rely on injections of artificial toxicants including NaIO_3_. Previously reported Dicer1-deficient mice [61, 62] could have served as a potential genetic mouse model for GA, but wider adoption in research laboratories has yet to be realized. GPx4 cKO mice demonstrate that the toxicity of lipid peroxidation products in the RPE can induce RPE degeneration similar to GA. Although lipid peroxidation in the RPE has long been considered a major pathogenic factor in AMD development [13–15], it has lacked direct in vivo evidence, especially for the late stage of dry AMD or GA.

GPx4 cKO mice exhibited key features resembling the late stage of dry AMD. Initially, RPE cells underwent a loss of polarized structures, including apical microvilli and basal infoldings. As degeneration progressed, we observed the loss of RPE cells, loss of photoreceptors, accumulation of lipofuscin, presence of subretinal/intraretinal melanophages, and activation of complement. Additionally, subretinal voids were observed in GPx4 cKO mice using both light microscopy (Supplementary Fig. S4) and TEM (Fig. 4D, F). Although the significance of these voids remains unclear, similar images have being reported in previous studies on AMD mouse models [40, 63]. Interestingly, extracellular lipid deposition beneath the RPE (resembling drusen) or in the subretinal space (resembling subretinal drusenoid deposits), characteristics of early/intermediate AMD, were not evident in GPx4 cKO mice. This absence may be attributed to the rapid degeneration of RPE cells, potentially limiting the time required for the gradual lipid deposition in the extracellular space. However, in the RPE-choroid of GPx4 cKO mice, we observed a significant accumulation of toxic lipid peroxidation products, including acrolein, MDA, and 4-HNE. Acrolein can be efficiently formed from PUFA [34], and is also a component of tobacco smoke, which is recognized as the most significant modifiable risk factor for AMD [35]. Additionally, acrolein is utilized in cellular models that mimics smoking-induced RPE cell death during AMD progression [36]. MDA and 4-HNE are constituents of lipofuscin in human RPE [37]. Recently, increased MDA levels in the RPE has been reported in a slowly progressive early/intermediate AMD model [38]. Furthermore, resent research has demonstrated the transport of intracellular lipids from RPE cells to the extracellular space, contributing to the formation of drusen, in a slowly progressive AMD model using a focused-ion beam scanning electron microscopy [42].

RPE degeneration in GPx4 cKO mice was alleviated by α-tocopherol (vit E) and Fer-1, accompanied by the observation of shrunk mitochondria, indicating the involvement of the ferroptotic mechanism. Interestingly, RPE degeneration was partially rescued by Nec-1s, suggesting the potential role of necroptosis in this process. Activation of necroptosis pathway was confirmed by presence of activated RIP3 and MLKL, along with inactivated caspase-8. We consider that GPx4 protects RPE cells from cell death by suppressing both ferroptosis and necroptosis pathways. While most studies have depicted GPx4 as a suppressor of ferroptosis, there are reports suggesting its involvement in inhibiting necroptosis in certain cell types. For example, a study demonstrated the inhibition of necroptosis by GPx4 in erythroid precursor cells [54]. Moreover, PUFA treatment on cancer cells induced lipid peroxidation-dependent necroptosis evidenced by elevated phosphorylation of RIP3 and MLKL, which was blocked by Fer-1 [64]. Furthermore, it has been shown that necroptosis and ferroptosis are alternative pathways in cultured fibroblasts. Deficiency of acyl-CoA synthetase long-chain family member 4 (ACSL4) results in resistance to ferroptosis but sensitivity to necroptosis, whereas deficiency of MLKL results in resistance to necroptosis as well as sensitivity to ferroptosis [65]. These findings suggest that the mechanisms for GPx4-mediated cell death prevention may vary depending on the cell types, and it is possible for necroptosis and ferroptosis to coexist. Further research will elucidate the molecular mechanisms underlying lipid peroxidation-induced RPE cell death.

After preprint publication of this manuscript, another report of GPx4 ablation in the RPE has recently been published [66]. The other study used tamoxifen-inducible Cre system to achieve GPx4 ablation in the adult mice. The data on RPE degeneration were in line with ours, while there are some differences. First, in our study the lipid peroxidation-dependent mechanism is demonstrated through a significant rescue effect by vitamin E and ferrostatin-1, while mechanistic investigations were not conducted in the other study. Second, the involvement of necroptosis is also discussed in our study. Third, the progression of RPE degeneration is faster in our model, while it takes months to achieve severe degeneration in the other study. This may be due to higher Cre expression levels in our AAV-Cre system, and our model may be convenient for the use in preclinical studies. Fourth, our study provides rigorous data on accompanying oxidative stress, complement activation, and infiltrating melaophages, while the other study largely depends on analyses of mRNA levels.

In conclusion, our current study unveiled the critical role of GPx4 in maintaining the homeostasis of RPE in mice. The synthesis of lipid peroxidation products in the RPE led to the manifestation of features resembling the late stage of dry AMD within a relatively short time period. GPx4 cKO mice offer a promising avenue to further explore the intricate relationship between lipid peroxidation and RPE degeneration. Additionally, they provide a valuable new option as a mouse model for preclinical research on GA.

## Supplementary information

Supplementary Information

Origincal western blot

## Data Availability

The published article includes all data sets generated/analyzed for this study.

## References

[1] Datta S, Cano M, Ebrahimi K, Wang L, Handa JT. The impact of oxidative stress and inflammation on RPE degeneration in non-neovascular AMD. Prog Retin Eye Res. 2017;60:201–18. 28336424 10.1016/j.preteyeres.2017.03.002 PMC5600827

[2] Strauss O. The Retinal Pigment Epithelium in Visual Function. Physiological Rev. 2005;85:845–81. 10.1152/physrev.00021.2004 15987797

[3] Wang W, Kini A, Wang Y, Liu T, Chen Y, Vukmanic E, et al. Metabolic Deregulation of the Blood-Outer Retinal Barrier in Retinitis Pigmentosa. Cell Rep. 2019;28:1323–34.e4. 31365873 10.1016/j.celrep.2019.06.093 PMC6693665

[4] Zhang Q, Presswalla F, Calton M, Charniga C, Stern J, Temple S, et al. Highly Differentiated Human Fetal RPE Cultures Are Resistant to the Accumulation and Toxicity of Lipofuscin-Like Material. Invest Ophthalmol Vis Sci. 2019;60:3468–79. 31408109 10.1167/iovs.19-26690 PMC6692057

[5] Saint-Geniez M, Kurihara T, Sekiyama E, Maldonado AE. D’Amore PA. An essential role for RPE-derived soluble VEGF in the maintenance of the choriocapillaris. Proc Natl Acad Sci USA. 2009;106:18751–6. 19841260 10.1073/pnas.0905010106 PMC2774033

[6] Young RW, Droz B. The renewal of protein in retinal rods and cones. J Cell Biol. 1968;39:169–84. 5692679 10.1083/jcb.39.1.169 PMC2107515

[7] Lewandowski D, Sander CL, Tworak A, Gao F, Xu Q, Skowronska-Krawczyk D. Dynamic lipid turnover in photoreceptors and retinal pigment epithelium throughout life. Prog Retin Eye Res. 2022;89:101037. 34971765 10.1016/j.preteyeres.2021.101037 PMC10361839

[8] Fu Z, Chen CT, Cagnone G, Heckel E, Sun Y, Cakir B, et al. Dyslipidemia in retinal metabolic disorders. EMBO Mol Med. 2019;11:e10473. 31486227 10.15252/emmm.201910473 PMC6783651

[9] Jin Z-B, Gao M-L, Deng W-L, Wu K-C, Sugita S, Mandai M, et al. Stemming retinal regeneration with pluripotent stem cells. Prog Retin Eye Res. 2019;69:38–56. 30419340 10.1016/j.preteyeres.2018.11.003

[10] Jager RD, Mieler WF, Miller JW. Age-related macular degeneration. N. Engl J Med. 2008;358:2606–17. 18550876 10.1056/NEJMra0801537

[11] Miller JW, Le Couter J, Strauss EC, Ferrara N. Vascular endothelial growth factor a in intraocular vascular disease. Ophthalmology. 2013;120:106–14. 23031671 10.1016/j.ophtha.2012.07.038

[12] Tzoumas N, Riding G, Williams MA, Steel DH. Complement inhibitors for age-related macular degeneration. Cochrane Database Syst Rev. 2023;6:CD009300. 37314061 10.1002/14651858.CD009300.pub3 PMC10266126

[13] Handa JT, Bowes Rickman C, Dick AD, Gorin MB, Miller JW, Toth CA, et al. A systems biology approach towards understanding and treating non-neovascular age-related macular degeneration. Nat Commun. 2019;10:3347. 31350409 10.1038/s41467-019-11262-1 PMC6659646

[14] Fleckenstein M, Keenan TDL, Guymer RH, Chakravarthy U, Schmitz-Valckenberg S, Klaver CC, et al. Age-related macular degeneration. Nat Rev Dis Prim. 2021;7:31. 33958600 10.1038/s41572-021-00265-2 PMC12878645

[15] Landowski M, Bowes Rickman C. Targeting Lipid Metabolism for the Treatment of Age-Related Macular Degeneration: Insights from Preclinical Mouse Models. J Ocul Pharm Ther. 2022;38:3–32. 10.1089/jop.2021.0067 PMC8817708 34788573

[16] Yang WS, SriRamaratnam R, Welsch ME, Shimada K, Skouta R, Viswanathan VS, et al. Regulation of Ferroptotic Cancer Cell Death by GPX4. Cell. 2014;156:317–31. 24439385 10.1016/j.cell.2013.12.010 PMC4076414

[17] Imai H, Hirao F, Sakamoto T, Sekine K, Mizukura Y, Saito M, et al. Early embryonic lethality caused by targeted disruption of the mouse PHGPx gene. Biochem Biophys Res Commun. 2003;305:278–86. 12745070 10.1016/s0006-291x(03)00734-4

[18] Imai H, Matsuoka M, Kumagai T, Sakamoto T, Koumura T. Lipid Peroxidation-Dependent Cell Death Regulated by GPx4 and Ferroptosis. In: Nagata S, Nakano H (eds). Apoptotic and Non-apoptotic Cell Death. Cham: Springer International Publishing; 2016, pp 143–70. 10.1007/82_2016_508 28204974

[19] Carlson BA, Tobe R, Yefremova E, Tsuji PA, Hoffmann VJ, Schweizer U, et al. Glutathione peroxidase 4 and vitamin E cooperatively prevent hepatocellular degeneration. Redox Biol. 2016;9:22–31. 27262435 10.1016/j.redox.2016.05.003 PMC4900515

[20] Friedmann Angeli JP, Schneider M, Proneth B, Tyurina YY, Tyurin VA, Hammond VJ, et al. Inactivation of the ferroptosis regulator Gpx4 triggers acute renal failure in mice. Nat Cell Biol. 2014;16:1180–91. 25402683 10.1038/ncb3064 PMC4894846

[21] Ueta T, Inoue T, Furukawa T, Tamaki Y, Nakagawa Y, Imai H, et al. Glutathione Peroxidase 4 Is Required for Maturation of Photoreceptor Cells. J Biol Chem. 2012;287:7675–82. 22207760 10.1074/jbc.M111.335174 PMC3293550

[22] Jia M, Qin D, Zhao C, Chai L, Yu Z, Wang W, et al. Redox homeostasis maintained by GPX4 facilitates STING activation. Nat Immunol. 2020;21:727–35. 32541831 10.1038/s41590-020-0699-0

[23] Wortmann M, Schneider M, Pircher J, Hellfritsch J, Aichler M, Vegi N, et al. Combined deficiency in glutathione peroxidase 4 and vitamin E causes multiorgan thrombus formation and early death in mice. Circ Res. 2013;113:408–17. 23770613 10.1161/CIRCRESAHA.113.279984

[24] Sano H, Kobayashi K, Yoshioka N, Takebayashi H, Nambu A. Retrograde gene transfer into neural pathways mediated by adeno-associated virus (AAV)-AAV receptor interaction. J Neurosci Methods. 2020;345:108887. 32739417 10.1016/j.jneumeth.2020.108887

[25] Yoo S-E, Chen L, Na R, Liu Y, Rios C, Van Remmen H, et al. Gpx4 ablation in adult mice results in a lethal phenotype accompanied by neuronal loss in brain. Free Radic Biol Med. 2012;52:1820–7. 22401858 10.1016/j.freeradbiomed.2012.02.043 PMC3341497

[26] Azuma K, Koumura T, Iwamoto R, Matsuoka M, Terauchi R, Yasuda S, et al. Mitochondrial glutathione peroxidase 4 is indispensable for photoreceptor development and survival in mice. J Biol Chem. 2022;298:101824. 35288190 10.1016/j.jbc.2022.101824 PMC8980337

[27] Xiong W, Wu DM, Xue Y, Wang SK, Chung MJ, Ji X, et al. AAV *cis* -regulatory sequences are correlated with ocular toxicity. Proc Natl Acad Sci USA. 2019;116:5785–94. 30833387 10.1073/pnas.1821000116 PMC6431174

[28] Afonso MB, Rodrigues PM, Carvalho T, Caridade M, Borralho P, Cortez-Pinto H, et al. Necroptosis is a key pathogenic event in human and experimental murine models of non-alcoholic steatohepatitis. Clin Sci. 2015;129:721–39. 10.1042/CS20140732 26201023

[29] Afonso MB, Rodrigues PM, Simão AL, Ofengeim D, Carvalho T, Amaral JD, et al. Activation of necroptosis in human and experimental cholestasis. Cell Death Dis. 2016;7:e2390–e2390. 27685634 10.1038/cddis.2016.280 PMC5059878

[30] Kanda Y. Investigation of the freely available easy-to-use software ‘EZR’ for medical statistics. Bone Marrow Transpl. 2013;48:452–8. 10.1038/bmt.2012.244 PMC3590441 23208313

[31] Iacovelli J, Zhao C, Wolkow N, Veldman P, Gollomp K, Ojha P, et al. Generation of Cre transgenic mice with postnatal RPE-specific ocular expression. Invest Ophthalmol Vis Sci. 2011;52:1378–83. 21212186 10.1167/iovs.10-6347 PMC3101664

[32] Chen M, Kim L, Lu CW, Zeng H, Vollrath D. An efficient inducible RPE-Selective cre transgenic mouse line. Exp Eye Res. 2021;202:108370. 33264655 10.1016/j.exer.2020.108370 PMC7856207

[33] Kocherlakota S, Baes M. Benefits and Caveats in the Use of Retinal Pigment Epithelium-Specific Cre Mice. Int J Mol Sci. 2024;25:1293. 38279294 10.3390/ijms25021293 PMC10816505

[34] Pan J, Chung F-L. Formation of cyclic deoxyguanosine adducts from omega-3 and omega-6 polyunsaturated fatty acids under oxidative conditions. Chem Res Toxicol. 2002;15:367–72. 11896684 10.1021/tx010136q

[35] DeAngelis MM, Ji F, Kim IK, Adams S, Capone A, Ott J, et al. Cigarette smoking, CFH, APOE, ELOVL4, and risk of neovascular age-related macular degeneration. Arch Ophthalmol. 2007;125:49–54. 17210851 10.1001/archopht.125.1.49

[36] Cui Y, Li Y, Huang N, Xiong Y, Cao R, Meng L, et al. Structure based modification of chalcone analogue activates Nrf2 in the human retinal pigment epithelial cell line ARPE-19. Free Radic Biol Med. 2020;148:52–59. 31887452 10.1016/j.freeradbiomed.2019.12.033

[37] Schutt F, Bergmann M, Holz FG, Kopitz J. Proteins modified by malondialdehyde, 4-hydroxynonenal, or advanced glycation end products in lipofuscin of human retinal pigment epithelium. Invest Ophthalmol Vis Sci. 2003;44:3663–8. 12882821 10.1167/iovs.03-0172

[38] Gupta U, Ghosh S, Wallace CT, Shang P, Xin Y, Nair AP, et al. Increased LCN2 (lipocalin 2) in the RPE decreases autophagy and activates inflammasome-ferroptosis processes in a mouse model of dry AMD. Autophagy. 2023;19:92–111. 35473441 10.1080/15548627.2022.2062887 PMC9809950

[39] Augustin S, Lam M, Lavalette S, Verschueren A, Blond F, Forster V, et al. Melanophages give rise to hyperreflective foci in AMD, a disease-progression marker. J Neuroinflammation. 2023;20:28. 36755326 10.1186/s12974-023-02699-9 PMC9906876

[40] Luhmann UFO, Robbie S, Munro PMG, Barker SE, Duran Y, Luong V, et al. The Drusenlike Phenotype in Aging *Ccl2* -Knockout Mice Is Caused by an Accelerated Accumulation of Swollen Autofluorescent Subretinal Macrophages. Invest Ophthalmol Vis Sci. 2009;50:5934. 19578022 10.1167/iovs.09-3462 PMC2801148

[41] Ban N, Lee TJ, Sene A, Choudhary M, Lekwuwa M, Dong Z, et al. Impaired monocyte cholesterol clearance initiates age-related retinal degeneration and vision loss. JCI Insight. 2018;3:e120824. 120824 30185655 10.1172/jci.insight.120824 PMC6171801

[42] Chuang J-Z, Yang N, Nakajima N, Otsu W, Fu C, Yang HH, et al. Retinal pigment epithelium-specific CLIC4 mutant is a mouse model of dry age-related macular degeneration. Nat Commun. 2022;13:374. 35042858 10.1038/s41467-021-27935-9 PMC8766482

[43] Xu H, Chen M, Manivannan A, Lois N, Forrester JV. Age‐dependent accumulation of lipofuscin in perivascular and subretinal microglia in experimental mice. Aging Cell. 2008;7:58–68. 17988243 10.1111/j.1474-9726.2007.00351.x

[44] Jaffe GJ, Westby K, Csaky KG, Monés J, Pearlman JA, Patel SS, et al. C5 Inhibitor Avacincaptad Pegol for Geographic Atrophy Due to Age-Related Macular Degeneration: A Randomized Pivotal Phase 2/3 Trial. Ophthalmology. 2021;128:576–86. 32882310 10.1016/j.ophtha.2020.08.027

[45] Armento A, Ueffing M, Clark SJ. The complement system in age-related macular degeneration. Cell Mol Life Sci. 2021;78:4487–505. 33751148 10.1007/s00018-021-03796-9 PMC8195907

[46] Mulfaul K, Ozaki E, Fernando N, Brennan K, Chirco KR, Connolly E, et al. Toll-like Receptor 2 Facilitates Oxidative Damage-Induced Retinal Degeneration. Cell Rep. 2020;30:2209–24.e5. 32075760 10.1016/j.celrep.2020.01.064 PMC7179253

[47] Tschopp J, Amiguet P, Schäfer S. Increased hemolytic activity of the trypsin-cleaved ninth component of complement. Mol Immunol. 1986;23:57–62. 3960033 10.1016/0161-5890(86)90171-9

[48] Otasevic V, Vucetic M, Grigorov I, Martinovic V, Stancic A. Ferroptosis in Different Pathological Contexts Seen through the Eyes of Mitochondria. Oxid Med Cell Longev. 2021;2021:5537330. 34211625 10.1155/2021/5537330 PMC8205588

[49] Ni L, Yuan C, Wu X. Targeting ferroptosis in acute kidney injury. Cell Death Dis. 2022;13:182. 35210424 10.1038/s41419-022-04628-9 PMC8873203

[50] Yuk H, Abdullah M, Kim D-H, Lee H, Lee S-J. Necrostatin-1 Prevents Ferroptosis in a RIPK1- and IDO-Independent Manner in Hepatocellular Carcinoma. Antioxid (Basel). 2021;10:1347. 10.3390/antiox10091347 PMC8469492 34572979

[51] Li J, Liu X, Liu Y, Huang F, Liang J, Lin Y, et al. Saracatinib inhibits necroptosis and ameliorates psoriatic inflammation by targeting MLKL. Cell Death Dis. 2024;15:122. 38331847 10.1038/s41419-024-06514-y PMC10853205

[52] Weber K, Roelandt R, Bruggeman I, Estornes Y, Vandenabeele P. Nuclear RIPK3 and MLKL contribute to cytosolic necrosome formation and necroptosis. Commun Biol. 2018;1:6. 30271893 10.1038/s42003-017-0007-1 PMC6123744

[53] Weinlich R, Oberst A, Beere HM, Green DR. Necroptosis in development, inflammation and disease. Nat Rev Mol Cell Biol. 2017;18:127–36. 27999438 10.1038/nrm.2016.149

[54] Canli Ö, Alankuş YB, Grootjans S, Vegi N, Hültner L, Hoppe PS, et al. Glutathione peroxidase 4 prevents necroptosis in mouse erythroid precursors. Blood. 2016;127:139–48. 26463424 10.1182/blood-2015-06-654194 PMC4705604

[55] Malek G, Johnson LV, Mace BE, Saloupis P, Schmechel DE, Rickman DW, et al. Apolipoprotein E allele-dependent pathogenesis: a model for age-related retinal degeneration. Proc Natl Acad Sci USA. 2005;102:11900–5. 16079201 10.1073/pnas.0503015102 PMC1187976

[56] Choudhary M, Ismail EN, Yao P-L, Tayyari F, Radu RA, Nusinowitz S, et al. LXRs regulate features of age-related macular degeneration and may be a potential therapeutic target. JCI Insight. 2020;5:e131928. 31829999 10.1172/jci.insight.131928 PMC7030875

[57] Imamura Y, Noda S, Hashizume K, Shinoda K, Yamaguchi M, Uchiyama S, et al. Drusen, choroidal neovascularization, and retinal pigment epithelium dysfunction in SOD1-deficient mice: a model of age-related macular degeneration. Proc Natl Acad Sci USA. 2006;103:11282–7. 16844785 10.1073/pnas.0602131103 PMC1544079

[58] Justilien V, Pang J-J, Renganathan K, Zhan X, Crabb JW, Kim SR, et al. SOD2 knockdown mouse model of early AMD. Invest Ophthalmol Vis Sci. 2007;48:4407–20. 17898259 10.1167/iovs.07-0432 PMC6549721

[59] Zhao Z, Xu P, Jie Z, Zuo Y, Yu B, Soong L, et al. γδ T cells as a major source of IL-17 production during age-dependent RPE degeneration. Invest Ophthalmol Vis Sci. 2014;55:6580–9. 25212781 10.1167/iovs.14-15166 PMC4203278

[60] Felszeghy S, Viiri J, Paterno JJ, Hyttinen JMT, Koskela A, Chen M, et al. Loss of NRF-2 and PGC-1α genes leads to retinal pigment epithelium damage resembling dry age-related macular degeneration. Redox Biol. 2019;20:1–12. 30253279 10.1016/j.redox.2018.09.011 PMC6156745

[61] Wright CB, Uehara H, Kim Y, Yasuma T, Yasuma R, Hirahara S, et al. Chronic Dicer1 deficiency promotes atrophic and neovascular outer retinal pathologies in mice. Proc Natl Acad Sci USA. 2020;117:2579–87. 31964819 10.1073/pnas.1909761117 PMC7007521

[62] Kaneko H, Dridi S, Tarallo V, Gelfand BD, Fowler BJ, Cho WG, et al. DICER1 deficit induces Alu RNA toxicity in age-related macular degeneration. Nature. 2011;471:325–30. 21297615 10.1038/nature09830 PMC3077055

[63] Hollyfield JG, Bonilha VL, Rayborn ME, Yang X, Shadrach KG, Lu L, et al. Oxidative damage-induced inflammation initiates age-related macular degeneration. Nat Med. 2008;14:194–8. 18223656 10.1038/nm1709 PMC2748836

[64] Suda A, Umaru BA, Yamamoto Y, Shima H, Saiki Y, Pan Y, et al. Polyunsaturated fatty acids-induced ferroptosis suppresses pancreatic cancer growth. Sci Rep. 2024;14:4409. 38388563 10.1038/s41598-024-55050-4 PMC10884029

[65] Müller T, Dewitz C, Schmitz J, Schröder AS, Bräsen JH, Stockwell BR, et al. Necroptosis and ferroptosis are alternative cell death pathways that operate in acute kidney failure. Cell Mol Life Sci. 2017;74:3631–45. 28551825 10.1007/s00018-017-2547-4 PMC5589788

[66] Wojciechowski AM, Bell BA, Song Y, Anderson BD, Conomikes A, Petruconis C, et al. Inducible RPE-specific GPX4 knockout causes oxidative stress and retinal degeneration with features of age-related macular degeneration. Exp Eye Res. 2024;247:110028. 39128667 10.1016/j.exer.2024.110028 PMC11392608

