# Supplementary material for: Retinal pigment epithelium-specific ablation of GPx4 in adult mice recapitulates key features of geographic atrophy in age-related macular degeneration
Source: Cell Death Dis. 2024 Oct 19;15(10):763. doi: 10.1038/s41419-024-07150-2 (PMC11490617; doi:10.1038/s41419-024-07150-2)
Supplement: Supplementary file 1 — Supplementary Information [file 41419_2024_7150_MOESM1_ESM.docx]

**Supplementary Information**

**Table S1. Antibodies used in this study.**

| **Antigen** | **Company** | **Catalogue#** | **Dilution (immunofluorescence)** | **Dilution (western blotting)** |
| --- | --- | --- | --- | --- |
| RIP3 | NOVUS | NBP1-77299 |  | 1:1000 |
| MLKL | Sigma-Aldrich | SAB1302339 |  | 1:500 |
| p-MLKL | Abcam | ab196436 |  | 1:1000 |
| Acrolein | NOF | N213320 |  | 1:1000 |
| 4-HNE | Abcam | Ab46545 | 1:2000 |  |
| GPx4 | established by Imai H. |  | 1:1000 | 1:5000 |
| GAPDH | Wako | 016-25523 |  | 1:1000 for insoluble fraction, 1:10000 for other samples |
| ZO-1 | Thermo Fisher Scientific | 61-7300 | 1:400 |  |
| C5b-9 | Santa-Cruz | sc-66190 |  | 1:600 |
| C3b/iC3b/C3c | Hycult | HM-1065 | 1:50 |  |
| Iba1 | wako | 019-19741 | 1:500 |  |
| F4/80 | Biorad | MCA-497 | 1:100 |  |
| cleaved-caspase 8 | Cell Signaling Technology | 8592 |  | 1:1000 |
| Cre | Merck | MAB3120 | 1:1000 |  |
| Anti-mouse IgG, HRP-linked Antibody | Cell Signaling Technology | 7076S |  | 1:3000~1:6000 |
| Anti-rabbit IgG, HRP-linked Antibody | Cell Signaling Technology | 7074S |  | 1:3000~1:6000 |
| Anti-rabbit Alexa Fluor 647 | Invitrogen | A32733 | 1:500 |  |
| Anti-mouse Alexa Fluor 647 | Invitrogen | A-21235 | 1:500 |  |
| Anti-rat Alexa Fluor 647 | Invitrogen | A-21247 | 1:500 |  |
| Anti-rabbit Alexa Fluor 488 | Invitrogen | A-11008 | 1:250 |  |

**Fig. S1**


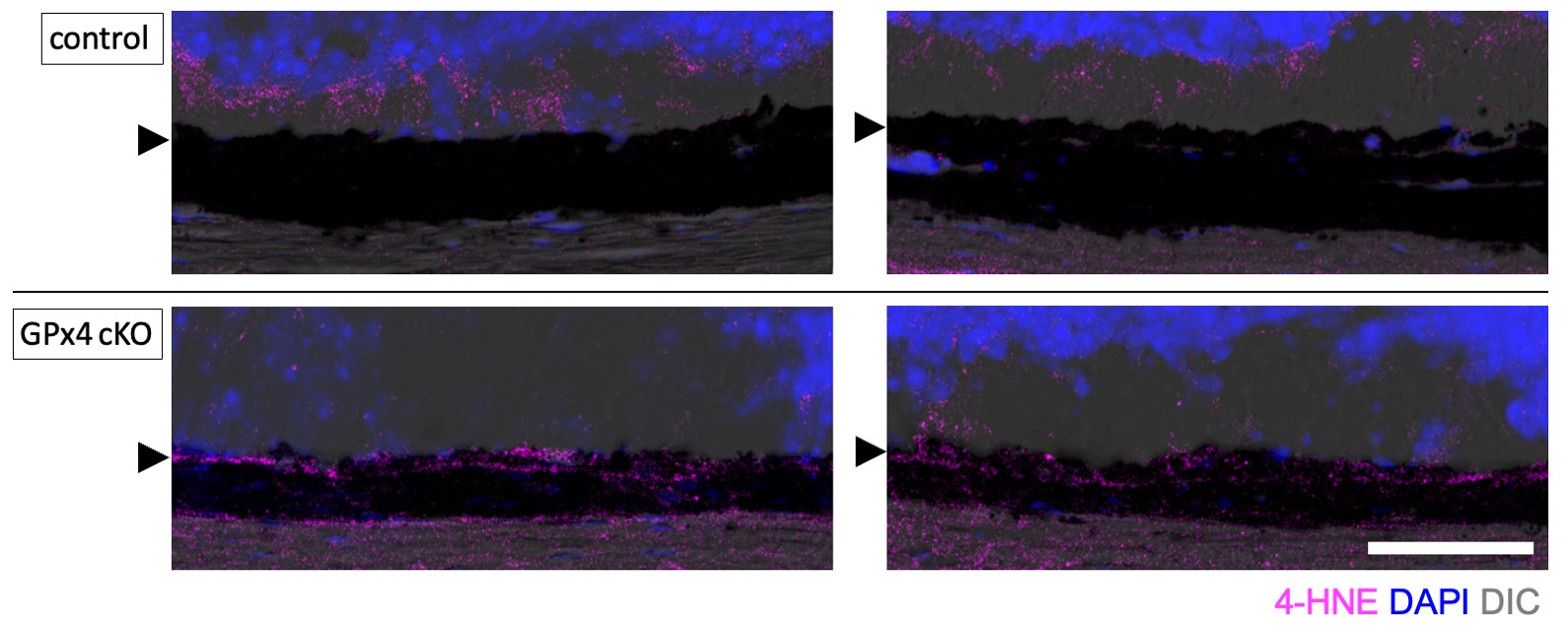


Additional two biological replicates for Fig. 1H. 4-HNE accumulation in the RPE was compared between GPx4 cKO and control mice after 12 days of subretinal AAV injections. Black arrowheads indicate RPE layer. Scale bar, 50µm.

**Fig. S2**

**
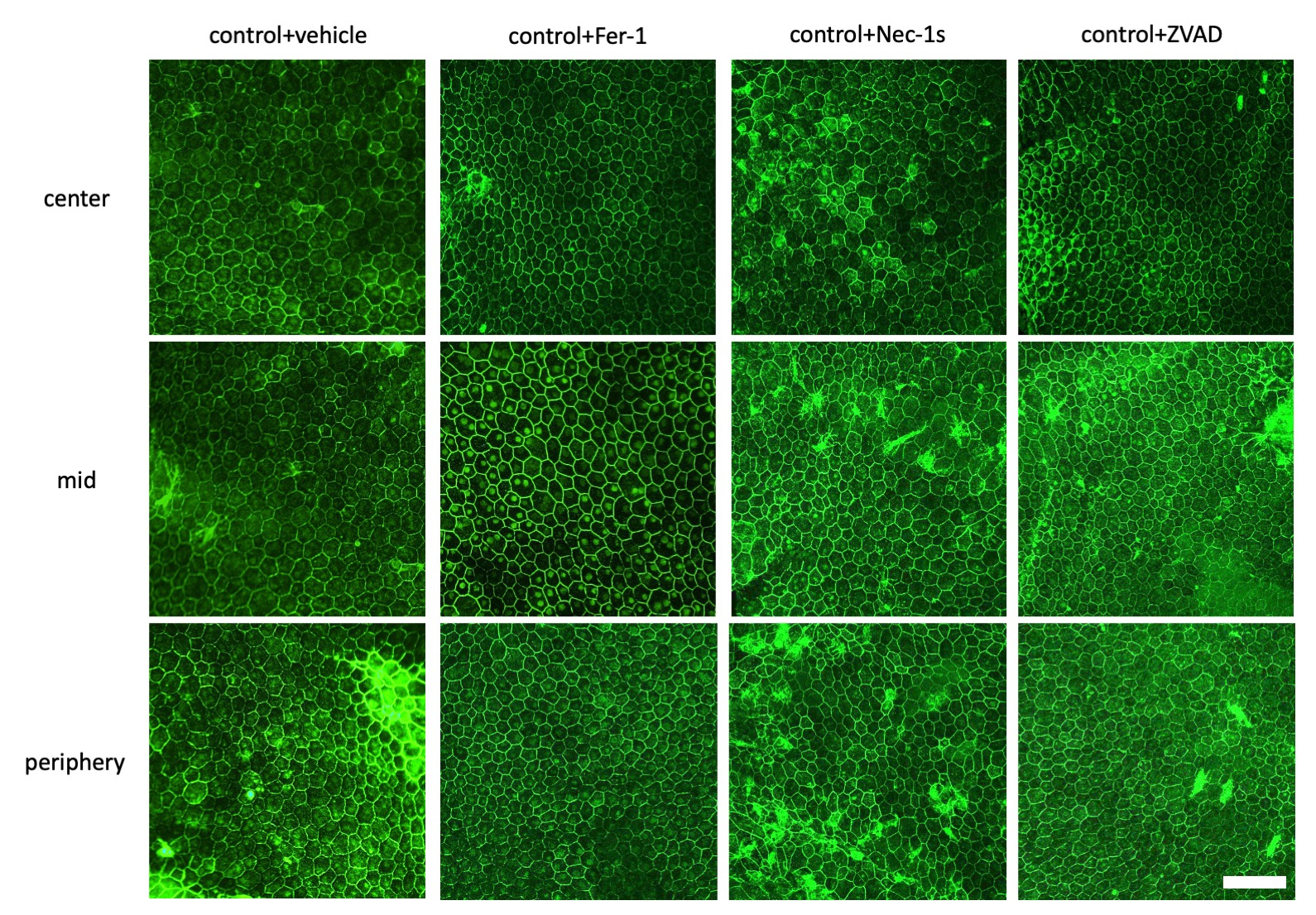
**

Fer-1, Nec-1s, ZVAD, or vehicle treatments did not affect RPE morphology in control mice 14 days after control AAV injections. Scale bar, 100µm.

**Fig. S3**


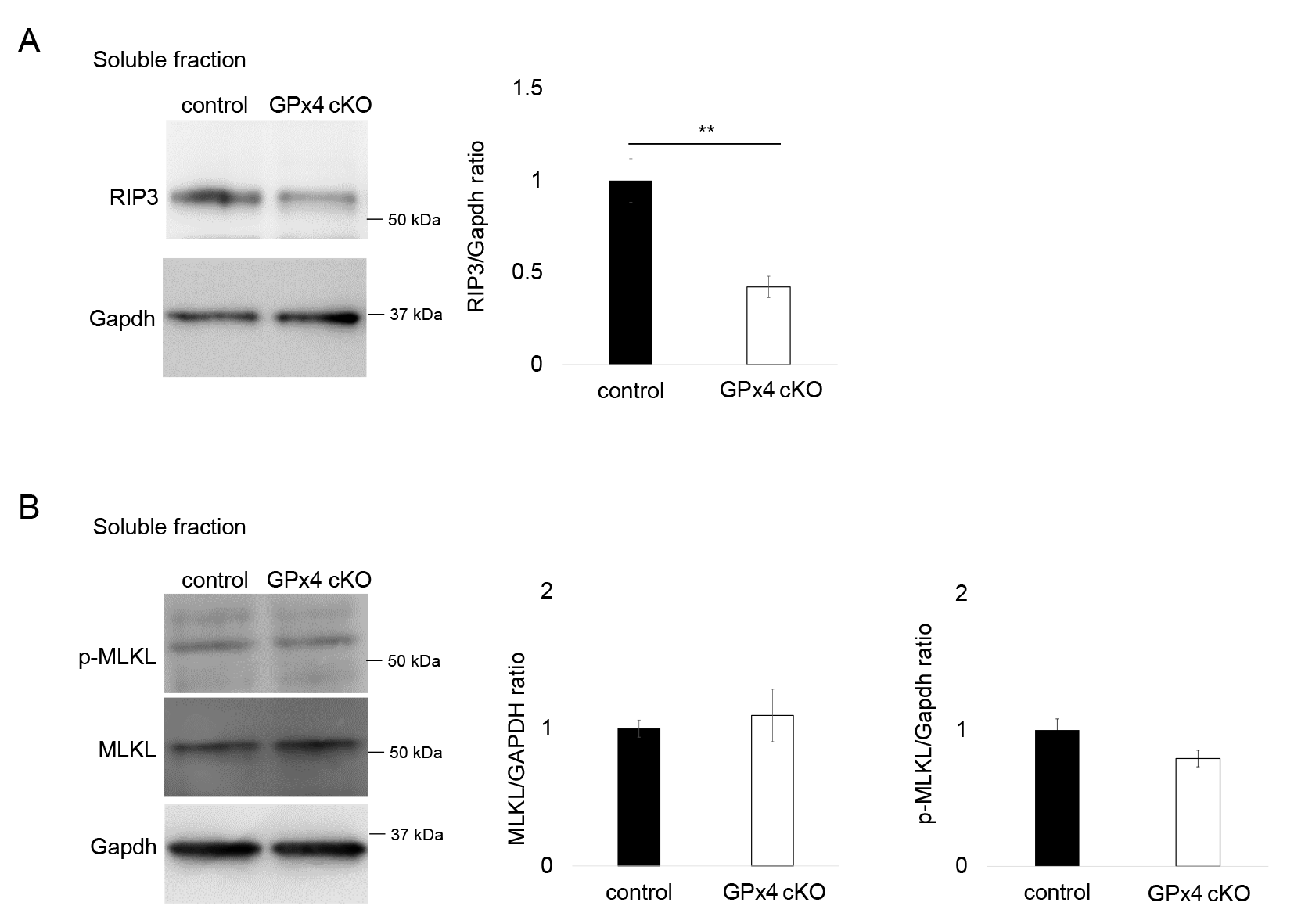


Abundance of (**A**) RIP3, (**B**) MLKL, and p-MLKL in detergent-soluble fraction samples of RPE-choroid was compared on western blot between GPx4 cKO and control mice 12 days after AAV injections. n=4 per group. There was no statistical significance in the levels of MLKL and p-MLKL between the two groups.

**Fig. S4**


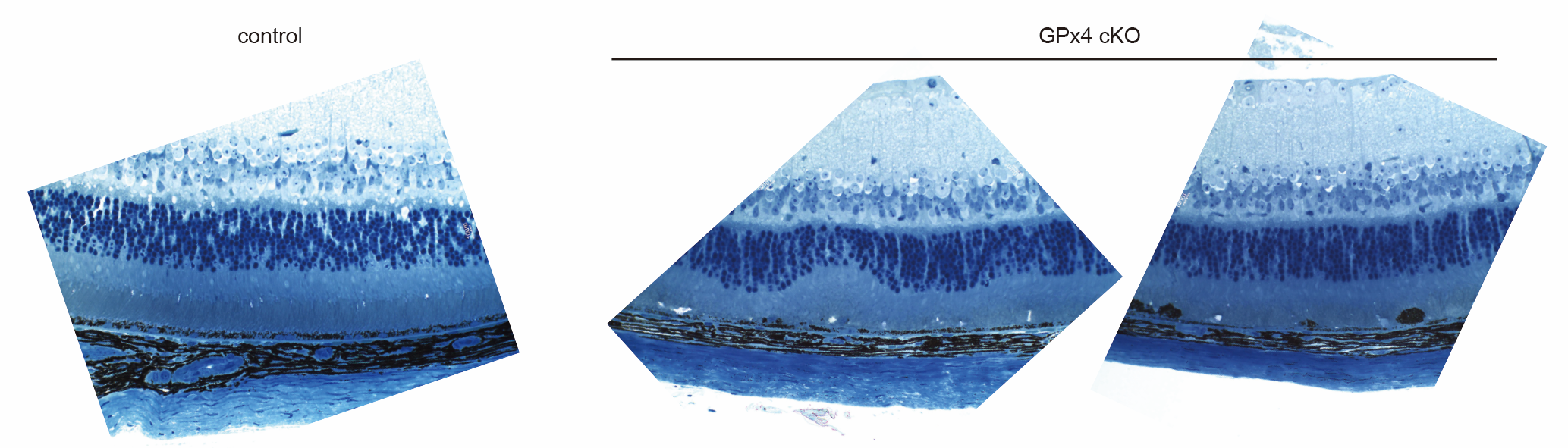


Toludine blue staining of the retina sections in GPx4 cKO and control mice 13 days after AAV injections. Note the multiple voids in the subretinal space of GPx4 cKO mice.
