# Supplementary figures and images for: Retinal pigment epithelium-specific ablation of GPx4 in adult mice recapitulates key features of geographic atrophy in age-related macular degeneration
Source: Cell Death Dis. 2024 Oct 19;15(10):763. doi: 10.1038/s41419-024-07150-2 (PMC11490617; doi:10.1038/s41419-024-07150-2)

Fig 1F

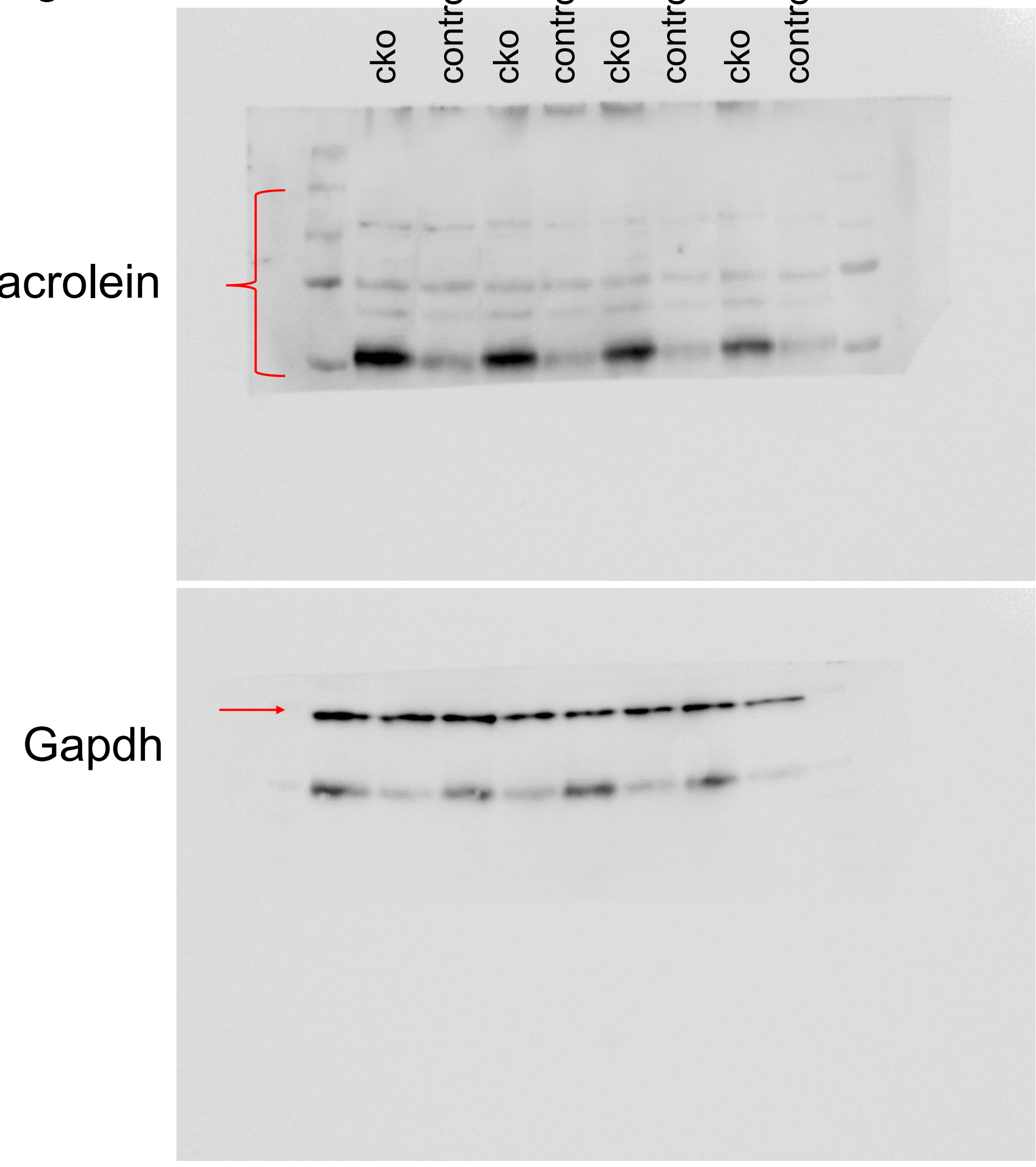

Fig 3H

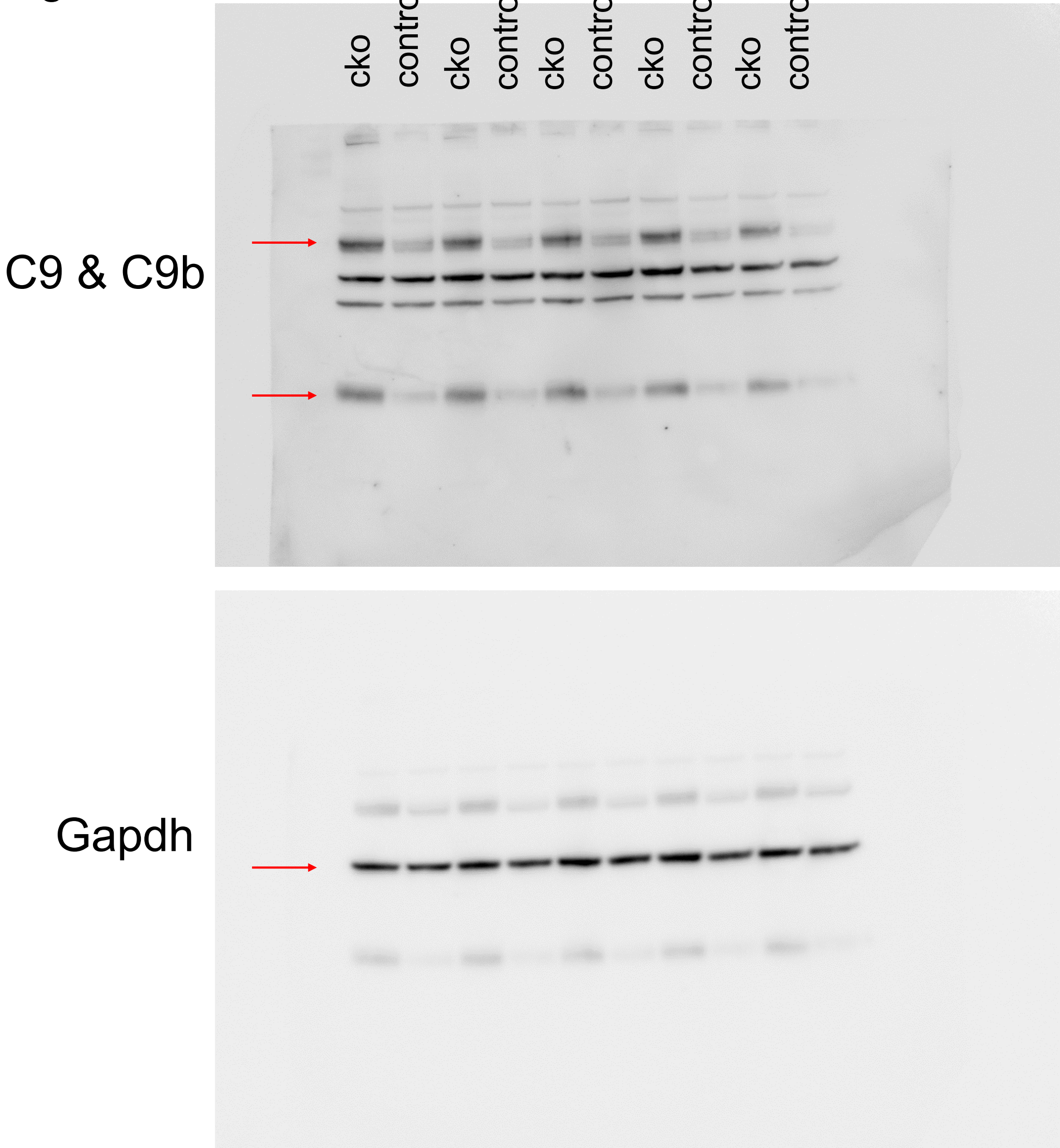

Fig S3A

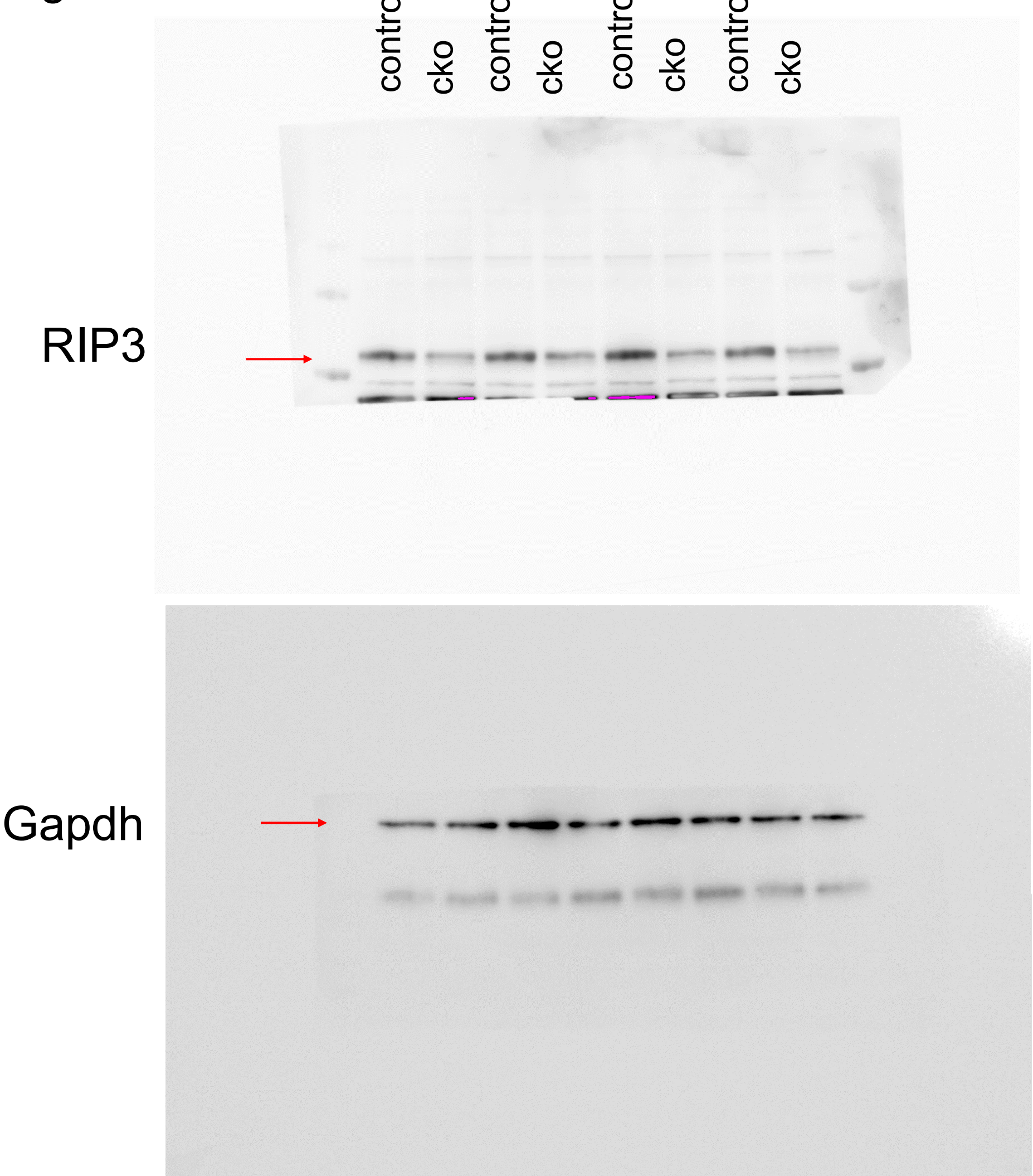

Fig 6B

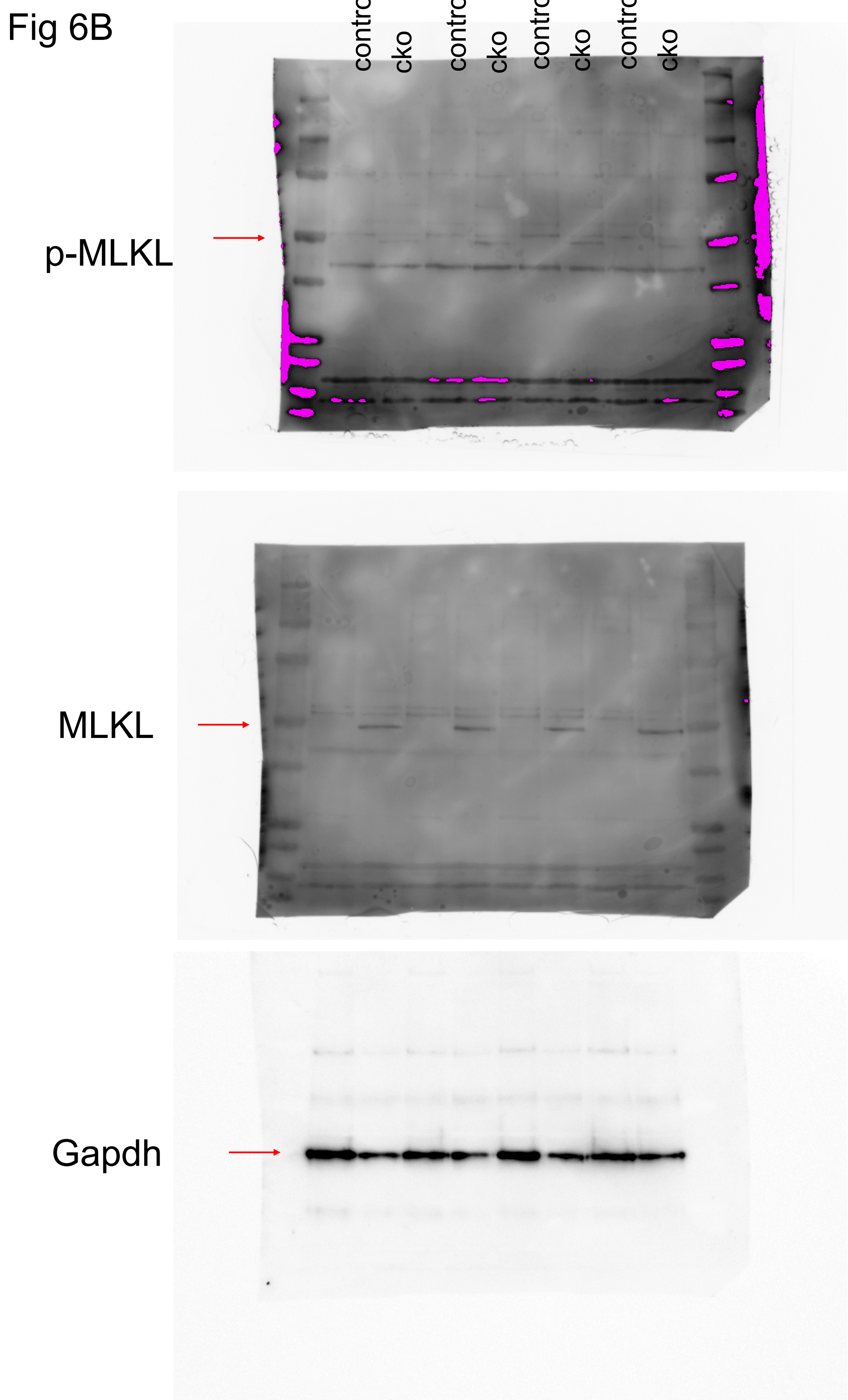

Fig 3H

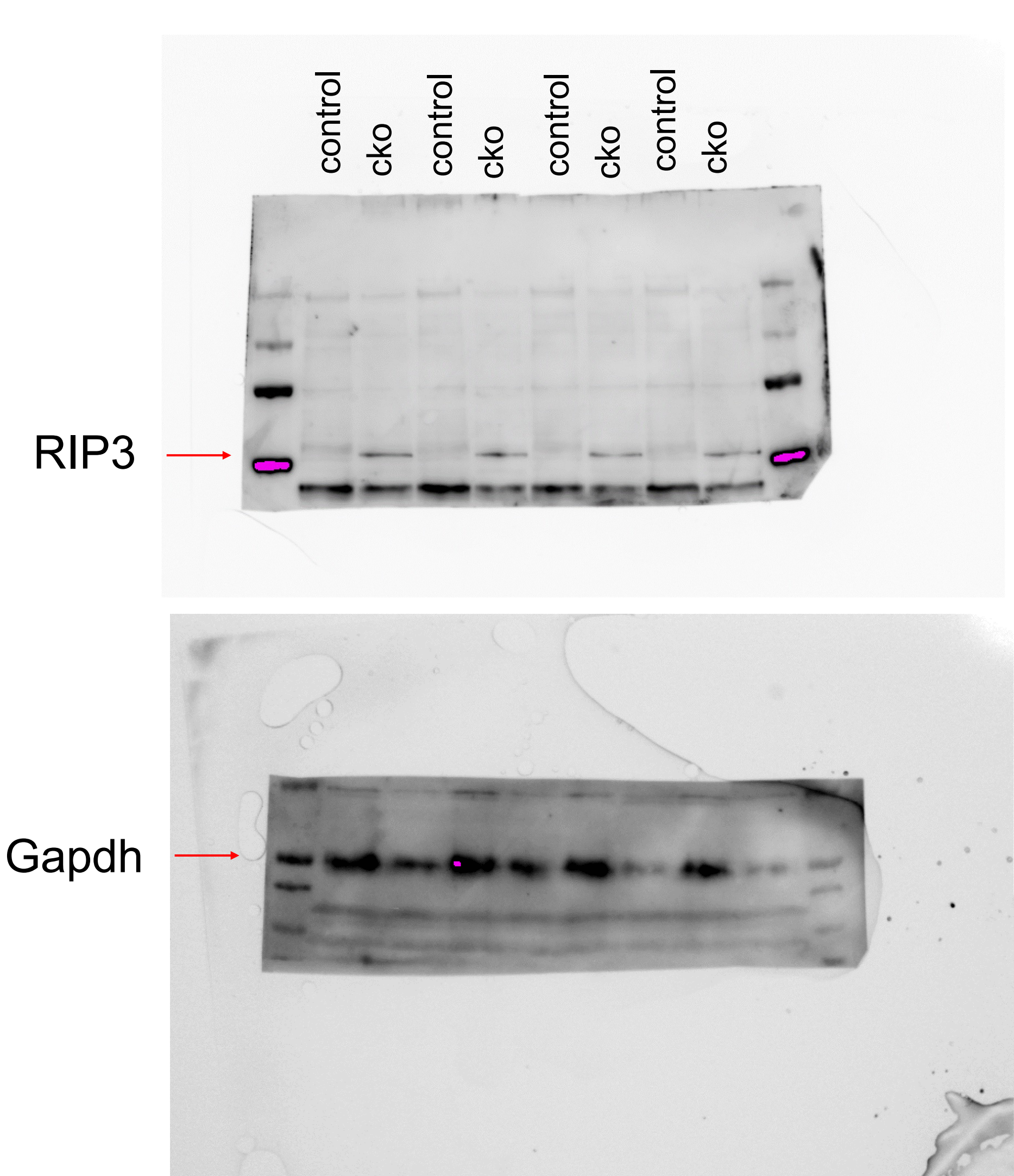

Fig S3B

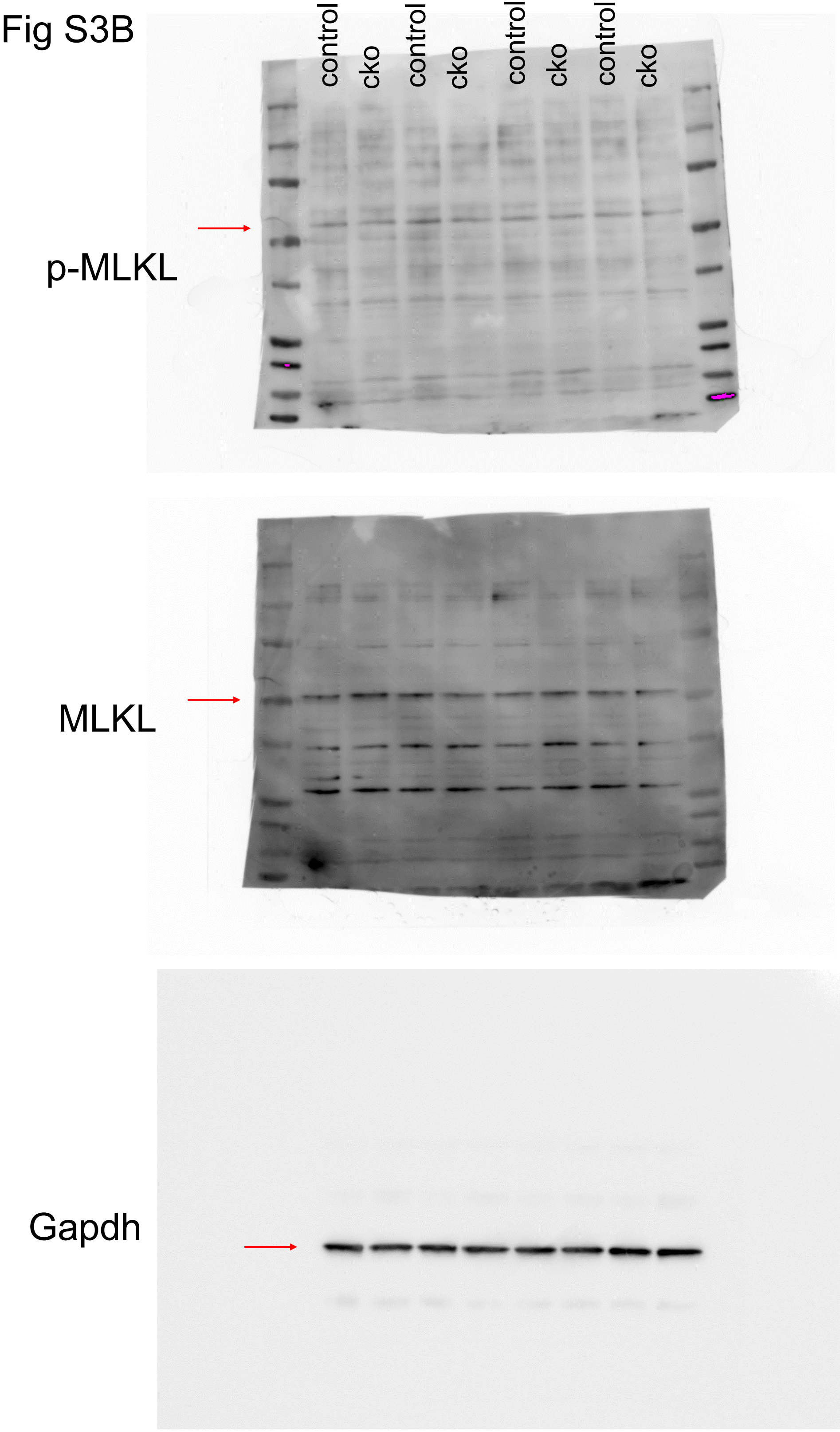

Fig 6C

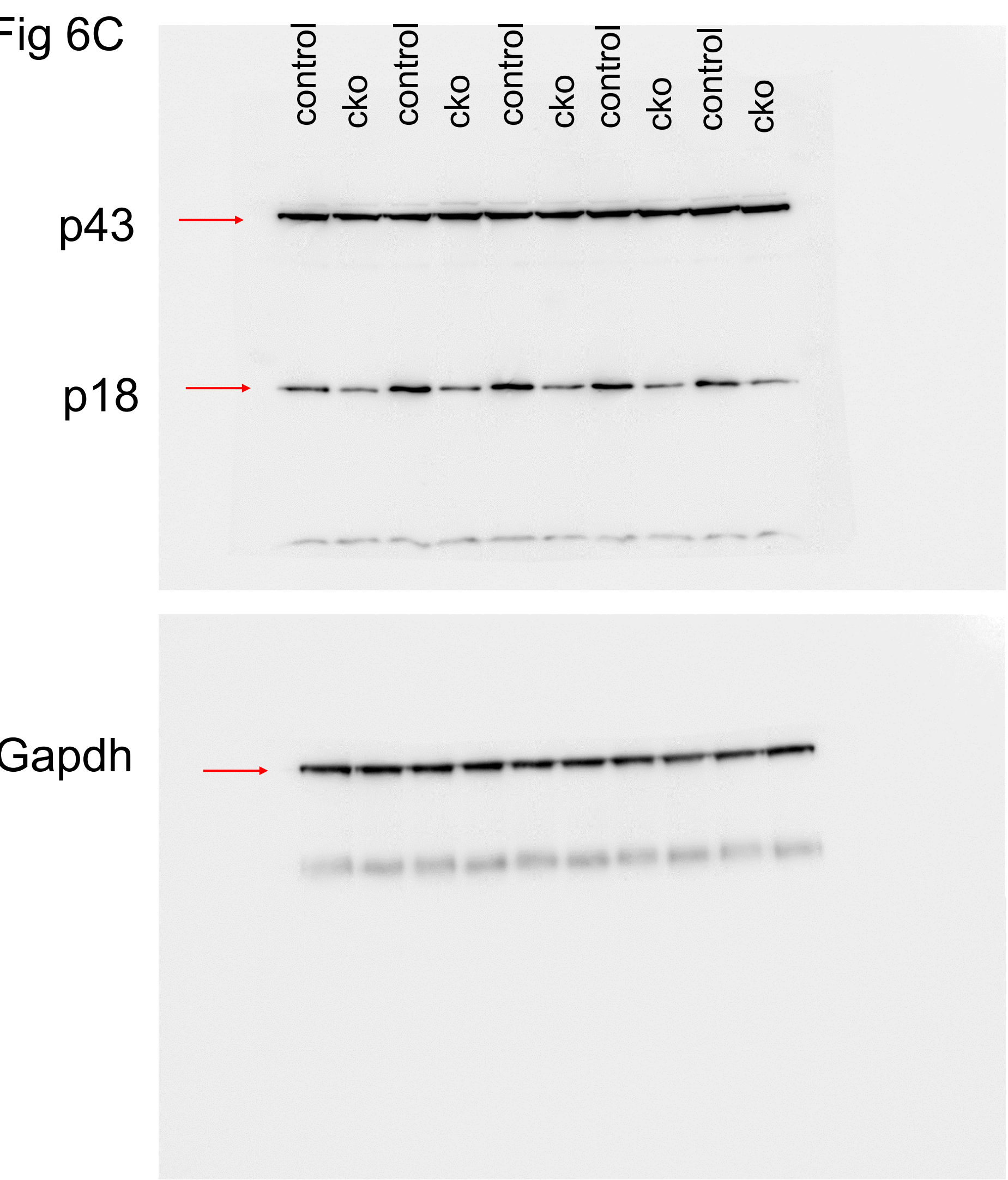

Supplement: Supplementary file 2 — Origincal western blot [file 41419_2024_7150_MOESM2_ESM.pdf]
